# Comprehensive analysis of the mitochondrial genome of *Iris domestica* emphasizing multichromosomal organization and repeat-mediated homologous recombination

**DOI:** 10.3389/fpls.2024.1520033

**Published:** 2025-02-27

**Authors:** Lizhi Ouyang, Xinyu Li, Ruili Wang, Yixuan Chen, Shuo Wang, Jianfang Wang, Yelin Tian

**Affiliations:** ^1^ College of Landscape Architecture, Beijing University of Agriculture, Beijing, China; ^2^ Inner Mongolia Academy of Science and Technology, Inner Mongolia, China

**Keywords:** plant mitochondrial genome, *Iris domestica*, genomic structure, multichromosomal chromosome, homologous recombination

## Abstract

**Background:**

*Iris domestica* is a perennial herb valued for both its ornamental and medicinal properties. Despite its significance, no comprehensive analysis of its mitochondrial genome has been previously reported. Plant mitochondrial genomes are known for their large size, structural complexity, and frequent recombination events. This study aims to provide the first complete assembly and characterization of the mitochondrial genome of *I. domestica*, with a focus on its structure, gene content, repeat elements, and RNA editing sites.

**Results:**

We used GetOrganelle and Unicycler software to hybrid assemble Nanopore and Illumina data to obtain the mitochondrial genome of *I. domestica*. The mitochondrial genome of *I. domestica* consists of four contigs: contig1 (222,498 bp), contig2 (90,780 bp), contig3 (42,563 bp), and contig4 (39,247 bp). Two repeat sequences, R1 (7,784 bp) and R2 (3,519 bp), facilitate the conformation of three circular chromosomes, suggesting a complex multi-chromosomal structure. A total of 34 protein-coding genes, including 24 core genes and 10 non-core genes were identified. Analysis of tandem repeat elements revealed significant variability, with Chromosome 1 showing the highest diversity of SSRs and scattered repeats. Additionally, 20 homologous fragments were identified between the mitochondrial and chloroplast genomes, accounting for 1.10% of the mitochondrial genome. Phylogenetic analysis based on 24 conserved mitochondrial genes placed *I. domestica* in close relation to *Iris domestica* and *Crocus sativus*. Furthermore, 545 RNA editing sites were identified, with notable variations across genes, suggesting that RNA editing plays a significant role in regulating mitochondrial gene expression.

**Conclusion:**

The complete assembly of the *I. domestica* mitochondrial genome reveals a complex multichromosomal structure characterized by recombination events. The high number of RNA editing sites and the presence of transferred plastid DNA highlight the dynamic nature of the genome, contributing to its adaptability and evolution. These findings provide a genetic foundation into the plant’s medicinal properties, adaptive mechanisms, and potential for environmental resilience.

## 1 Introduction

*Belamcanda chinensis* belongs to the family Iridaceae (Garcia de la Garma et al., 2015). Taxonomically, it is currently classified within the genus *Iris*, though it was previously designated as *Iris domestica* (Goldblatt and Mabberley, 2005).

This perennial herbaceous plant is indigenous to East Asia, with its native range encompassing China, Japan, Korea, and Russia (Goldblatt and Mabberley, 2005). Over time, *I. domestica* has naturalized in various regions, including North America and Europe, largely due to its ornamental value (Buta et al., 2010; Xin et al., 2015). Globally, *I. domestica* is cultivated for both its aesthetic qualities—most notably its distinctive orange flowers adorned with red spots—and its medicinal applications (Larbie and Abboah-Offei, 2014). The plant is known to contain several bioactive compounds, including flavonoids, iridoids, and triterpenoids, which exhibit antimicrobial and antioxidant properties (Min-Jian et al., 2000; Jung et al., 2004; Xin et al., 2015; Ni et al., 2018; Kim et al., 2021). In traditional Chinese medicine, extracts derived from the roots and rhizomes of *I. domestica* have been employed in the treatment of a variety of ailments, such as sore throat, asthma, cough, and various infections (Chen et al., 2004; Hempen and Fischer, 2009; Gao et al., 2014; Wang et al., 2024). Furthermore, recent pharmacological studies suggest that the plant may possess anticancer properties, making it a promising candidate for further biomedical research (Jung et al., 2003; Monthakantirat et al., 2005; Liu et al., 2012a; Liu et al., 2012b). From an economic perspective, *I. domestica* is valuable due to its dual role in both horticulture and herbal medicine (Xin et al., 2015; Zhang et al., 2016; Wang et al., 2024). Its visual appeal ensures its popularity in gardens and landscaping projects, while its medicinal attributes provide substantial opportunities for the development of herbal remedies and pharmaceutical products (Singab et al., 2016; Patel and Kate, 2024). The ongoing cultivation and commercial exploitation of *I. domestica* therefore contribute significantly to the ornamental plant industry as well as the burgeoning market for herbal and natural medicines, underscoring its multifaceted economic importance.

The mitochondrial genome’s origin is attributed to an ancient endosymbiotic event in which a proteobacterial ancestor integrated into a eukaryotic host cell, resulting in the formation of a semi-autonomous organelle containing its own genetic material (Roger et al., 2017). The primary function of the mitochondrial genome is to encode proteins for ATP production, which processes that are central to cellular energy metabolism (Vakifahmetoglu-Norberg et al., 2017). Most land plant mitochondrial genomes exhibit remarkable structural diversity, characterized by their large size, the presence of extensive repetitive sequences, and a complex organization that often includes multipartite structures or circular DNA molecules (Mower et al., 2012b; Bi et al., 2024). Unlike many other eukaryotic genomes, plant mitochondrial genomes display a high degree of recombination, which is facilitated by these repetitive sequences (Arrieta-Montiel and Mackenzie, 2011; Gualberto et al., 2014). This recombination can lead to significant structural rearrangements and the formation of sub-genomic molecules, contributing to the dynamic nature (Gualberto et al., 2014). A distinctive feature is RNA editing, a post-transcriptional process in which specific nucleotides in RNA transcripts are altered, commonly changing cytidine to uridine. This modification is essential for producing functional proteins from the mitochondrial genome (Takenaka et al., 2008). Moreover, plant mitochondrial genomes are capable of horizontal gene transfer, wherein genetic sequences are transferred from the mitochondrial genome to the nuclear, chloroplast genome or between different plant species (Xiong et al., 2008; Sanchez-Puerta, 2014). Such gene transfer events contribute to genetic diversity and have significant implications for the evolution of mitochondrial function and plant adaptation (Mower et al., 2012a; Wang et al., 2018).

The mitochondrial genome of *I. domestica* is of significant importance due to its roles in energy metabolism and its influence on the plant’s overall physiological functions. This study is the first to report the complete mitochondrial genome of *I. domestica*, providing a comprehensive analysis of its repeat elements, structural diversity, recombination events, RNA editing processes, and sequence migration. These analyses offer detailed insights into the unique features of the *I. domestica* mitochondrial genome, laying a genetic foundation for further research into the plant’s medicinal properties, evolutionary biology, and potential for adaptation to various environmental stresses.

## 2 Materials and methods

### 2.1 DNA extraction and sequencing

Specimens of *I. domestica* were collected systematically from Beijing Agricultural College, located at a longitude of 116.31 and latitude of 40.0941. Immediately after collection, the leaves were carefully washed with DPEC water to ensure maximum sample purity. Genomic DNA was isolated using the Magnetic Plant Genomic DNA Kit provided by Tiangen, China. For short-read sequencing, a DNA library, with an average insert length of 350 bp, was constructed using the TIANSeq Fast DNA Library Kit (California, USA), followed by sequencing on the Illumina HiSeq X platform (California, USA). For long-read sequencing, DNA was extracted using the DNA Extraction Kit (New England Biolabs, Massachusetts, USA) to preserve high molecular weight DNA. A DNA library with an average fragment size of 10 kb was prepared with the DNA Library Kit (TIANGEN), and sequencing was performed using a PromethION sequencer (Beijing, China) incorporating Oxford Nanopore technology.

### 2.2 Organelle genome obtained

The hybrid methodology was adopted for the construction of the organelle genomes of *I. domestica*. Initial retrieval of targeted sequence reads from organelle genomes was accomplished through the GetOrganelle tool based on its default reference genome (*Vigna radiata*, NC_015121.1) (Jin et al., 2020), with default settings for mitochondria from Illumina sequencing outputs. These sequences were then assembled *de novo* within the Unicycler framework employing SPAdes (Bankevich et al., 2012), creating an extensive genome map. Double bifurcations within the map were addressed by aligning Nanopore long reads utilizing BWA software (Li and Durbin, 2009). Annotation of the chloroplast genome was executed on the CPGAVAS2 (Shi et al., 2019) and validated via the CPGView server (Liu et al., 2023), while the mitochondrial genome underwent annotation through the PMGA web server (Li et al., 2024), with visualization provided by the OGdraw web server (Greiner et al., 2019).

### 2.3 Repeat elements analysis and identification of mitochondrial plastid DNAs

In this investigation, we identified two distinct categories of tandem repeat elements within the genome: Simple Sequence Repeats (SSRs) and standard tandem repeats. The SSRs were detected utilizing the MISA with the parameters “1-10 2-5 3-4 4-3 5-3 6-3” and “Max. length of sequence between two SSRs to register as compound SSR = 0” (Beier et al., 2017), while the standard tandem repeats were ascertained through TRF with default parameters (Benson, 1999). The spatial organization and distribution of these repeat elements were visualized using the Circos tool within the TBtools software suite (Chen et al., 2020). Furthermore, intergenomic transfers of Mitochondrial Plastid DNAs (MTPTs) between the organelle genomes were detected using the BLASTn program with the parameters “evalue 1e-5, sequence similarity 80%” (Chen et al., 2015).

### 2.4 Phylogenetic analysis

Common genes from the mitochondrial genomes of *I. domestica* and its phylogenetically related species were meticulously extracted utilizing PhyloSuite software (version 1.1.16) (Zhang et al., 2020). Subsequent alignments of these gene sequences were concentrated and conducted employing MAFFT software (version 7.505) (Katoh and Standley, 2013). The phylogenetic reconstruction was performed via the maximum likelihood method, utilizing IQ-TREE software (version 1.6.12) (Nguyen et al., 2015) under the parameters “–alrt 1000 -B 1000” and the evolutionary model GTR+F+I+R2. This method included 1000 bootstrap iterations to assess the robustness of the phylogenetic inference. Finally, the derived phylogenetic relationships were effectively visualized using ITOL software (version 6) (Letunic and Bork, 2019), facilitating a clear representation of evolutionary lineage relationship.

### 2.5 RNA editing sites event identification

The identification of RNA editing sites was performed using the Deepred-mt software (Edera et al., 2021), which utilizes a convolutional neural network (CNN) model. We filtered results to include only predictions with probability scores exceeding 0.9.

## 3 Results

### 3.1 Assembly and structural analysis

**Figure 1:**
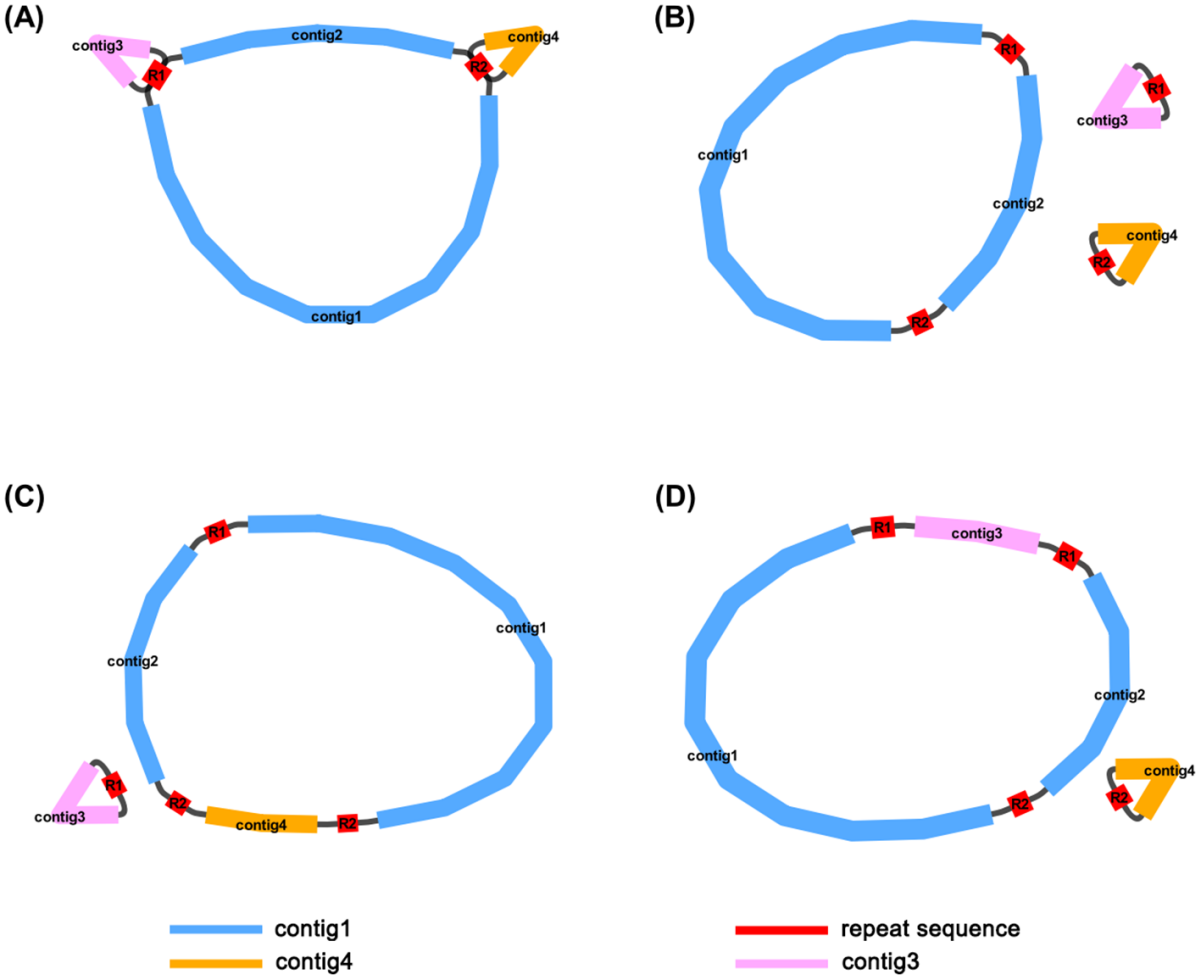
Mitochondrial Genome Structures of *I domestica*. **(A)** The complete, unprocessed graphical representation of the assembled mitochondrial genome, containing four contigs (contig1, contig2, contig3, and contig4) and two repeat sequences (R1 and R2). This structure represents the full assembly before circularization and resolution of the repeat sequences. **(B)** The primary structure showing three circular chromosomes that result from resolving the genome in panel **(A)**. Contig1 and contig2 form a larger circular chromosome, while contig3 and contig4 form two smaller individual circular chromosomes, with R1 and R2 marking the repeat boundaries. **(C)** An alternative conformation derived from **(A)**, consisting of two circular chromosomes. Contig1 and contig2 remain linked, while contig4 is now incorporated into the larger circle, and contig3 is left as a separate, smaller chromosome. **(D)** Another alternative conformation, also derived from **(A)**, with two circular chromosomes formed by a different arrangement. In this case, contig1 and contig2 remain connected, but contig3 is now incorporated into the larger circle, and contig4 forms the smaller chromosome. The blue lines represent contig1 and contig2, the orange line represents contig4, and the pink line represents contig3. The red lines represent repeat sequences (R1 and R2) that mediate homologous recombination, facilitating the formation of different mitochondrial conformations.

The dominant structure of the *I. domestica* mitochondrial genome is characterized by the branch structure. The branch structure is formed by the connection of multiple mitochondrial DNA molecules and represents an intermediate form of the mitochondrial genome. Based on the graphical assembly results, the mitochondrial genome of *I. domestica* consists of four contigs: contig1 (222,498 bp), contig2 (90,780 bp), contig3 (42,563 bp), and contig4 (39,247 bp). The repeat sequence R1 (7,784 bp) connects contig1, contig2, and contig3, while the repeat sequence R2 (3,519 bp) connects contig1 and contig4 (**Figure 1A**). Supported by read data, three primary circular chromosome conformations have been inferred (**Figure 1B**; **Table 1**). Chromosome 1 is composed of contig1, contig2, R1, and R2; Chromosome 2 consists of R1 and contig3; and Chromosome 3 is formed by R2 and contig4. Additionally, recombination events between R1 and R2 suggest two alternative double-chromosome conformations (**Figures 1C, D**), reflecting the structural complexity and recombination diversity of the mitochondrial genome. These assembly results provide a foundational framework for further investigation into the structure and function of the *I. domestica* mitochondrial genome.

**Table 1:** Basic information of the mitochondrial genome.

NCBI Accession number	Contigs	Type	Length (bp)	GC content (%)
PQ468093	Chromosome 1	circular	324581	46.51
PQ468094	Chromosome 2	circular	50343	47.66
PQ468095	Chromosome 3	circular	42766	47.56

### 3.2 Genome recombination analysis

**Figure 2:**
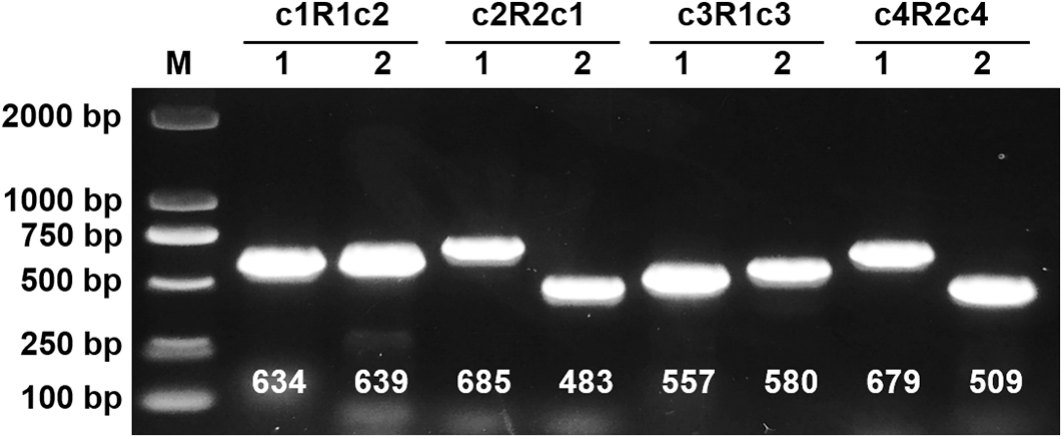
Mitochondrial Genome Reconbination Validation of *I. domestica*. Lane M contains a DNA ladder with markers indicated in base pairs (bp). Lanes 1 and 2 for configurations c1R1c2, c2R2c1, c3R1c3, and c4R2c4 display PCR amplified products, with expected sizes labeled below each band in base pairs. The numbers (634, 639, 685, 483, 557, 580, 679, 509) indicate the approximate size of the PCR products corresponding to each configuration.

To validate the proposed configurations, we initially employed long-read sequencing to map each configuration independently, subsequently quantifying the reads associated with each (**Table 2**). The recombinant sequence in question posits four potential genomic paths, achieved through an extension of 2,000 bp at both termini of a repetitive sequence. This extension resulted in a total mapping length of 11,784 bp for configuration R1 and its flanking sequences, and 7,519 bp for configuration R2 and its extensions. Analysis revealed that the configuration with the highest read support, totaling 13 reads, corresponds to three distinct circular chromosomes. In contrast, only four reads supported alternative configurations. Additionally, to confirm the integrity and existence of the repetitive sequence boundaries within each configuration, we conducted PCR amplification followed by Sanger sequencing at these specific regions (**Figure 2**; **Supplementary Files 1**-**8**). The results affirm that the triplet of independent circular chromosomal structures represents the dominant configuration, while the presence of other configurations, though verifiable, is comparatively minimal.

**Table 2:** Using long-reads to validate potential repeat-mediated recombinations in mitogenome based on BLASTn.

No.	Path	Reads ID	Identity (%)	Alignment (bp)	Reference start	Reference end	Reads start	Reads end
1	ctg1-R1-ctg2	562c7825-9e7e-4ee8-b743-629e0c5429e0	94.231	11,977	1	11,782	20,947	9,194
2	ctg1-R1-ctg2	1151f81d-0d93-4347-906d-d2a948e02643	97.768	11,603	252	11,784	26,709	15,213
3	ctg3-R1-ctg3	2286ff3b-1bcf-484e-8d90-a9f0fc049c2c	96.615	11,907	1	11,784	25,444	13,695
4	ctg3-R1-ctg3	3cc576dd-d502-4e1b-993e-5d1a8a2b664a	91.94	10,633	1,351	11,784	15,543	5,203
5	ctg3-R1-ctg3	b730da28-53be-40f1-8696-93ad05af9df7	92.45	9,695	761	10,352	1	9,210
6	ctg2-R2-ctg1	6c784803-80c8-418f-9875-8a61fa70a442	95.546	7,589	1	7,519	1,280	8,722
7	ctg2-R2-ctg1	2b3fb0ce-2925-43d1-b629-bdc159c94af4	94.706	5,837	1,089	6,858	1	5,718
8	ctg4-R2-ctg4	b23bc2dc-f634-42a7-a5a4-4fce6ea8ea2b	88.29	7,737	1	7,518	17,324	9,867
9	ctg4-R2-ctg4	45ed6141-ce33-459f-b9c0-7abb14d59bb9	84.447	7,722	26	7,519	21,420	14,093
10	ctg4-R2-ctg4	2652d0ea-c9ef-40fc-8afb-00c9138a3b8f	92.389	7,660	1	7,519	8,225	772
11	ctg4-R2-ctg4	c620907e-5bd2-4d9d-b0e7-1e0c9caaa892	92.478	7,658	1	7,519	8,512	1,055
12	ctg4-R2-ctg4	0edb2542-3667-427c-a802-8bc9fe1fc5e3	97.11	7,578	1	7,519	8,470	15,959
13	ctg4-R2-ctg4	562afc1c-6b1f-47d5-a0a8-d376cd34e362	96.808	7,363	213	7,519	18,473	25,742
14	ctg2-R2-ctg4	c4604840-5506-41df-921a-1625836b610a	97.676	7,572	1	7,519	10,027	2,525
15	ctg2-R2-ctg4	81dc97e4-9b4f-42f5-a6bc-3ed9c41cce8a	96.139	7,433	1	7,375	7,336	6
16	ctg2-R2-ctg4	804e0dd3-25c6-40ff-b219-53ea20270aa7	88.613	6,077	1,235	7,159	5,887	1
17	ctg4-R2-ctg1	5da75316-8aa7-455b-8a7e-0b24b33339f6	89.496	7,711	1	7,519	56,423	49,073

### 3.3 Gene and repeat annotation

**Figure 3:**
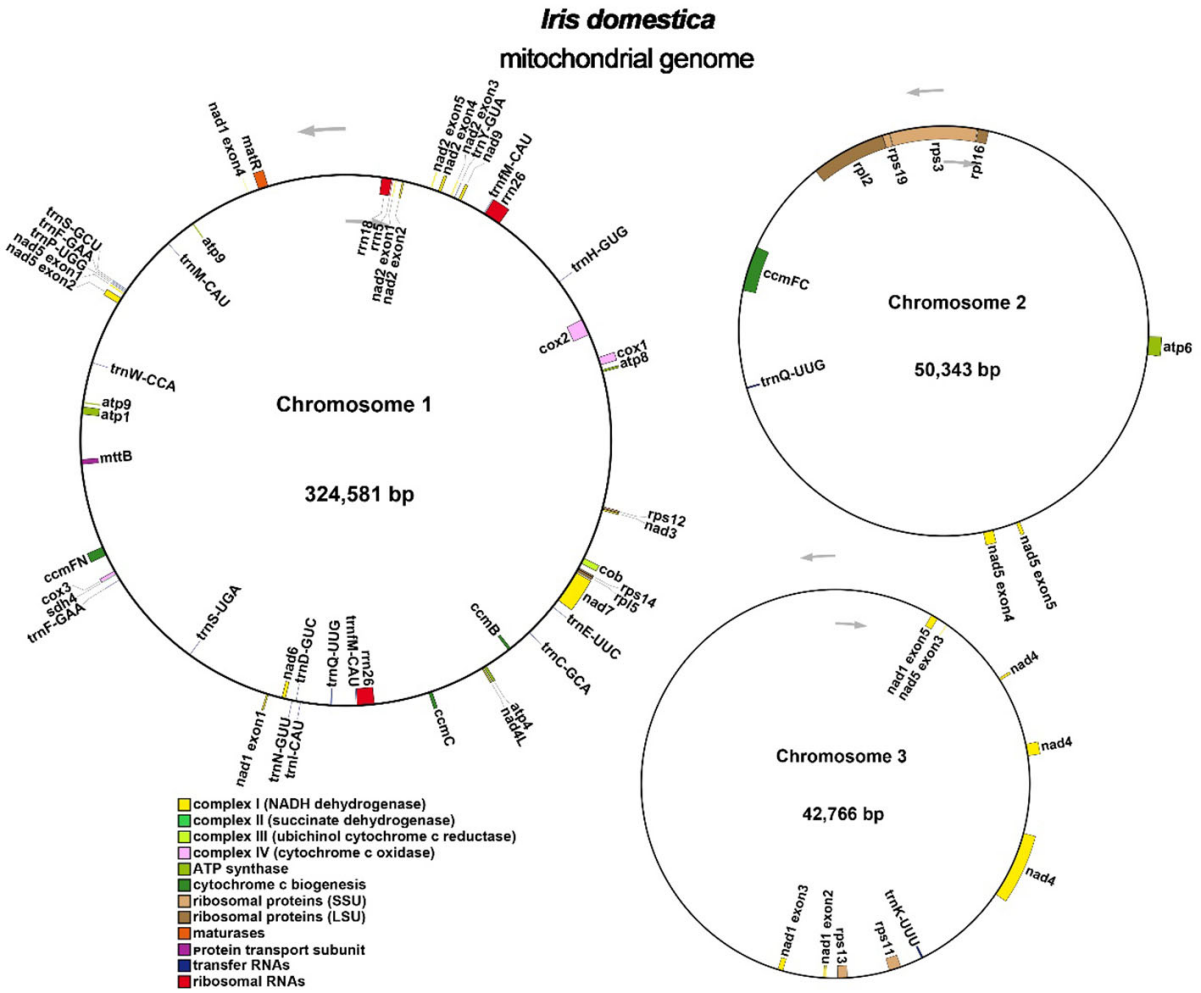
Organization of the *I. domestica* Mitochondrial Genome Across Three Chromosomes. The mitochondrial genome is depicted across three chromosomes. Genes are annotated and color-coded based on functional categories: Complex I (NADH dehydrogenase) in yellow, Complex III (succinate dehydrogenase) in dark green, Complex IV (cytochrome c oxidase) in blue, ATP synthase in purple, cytochrome c biogenesis complex in orange, ribosomal proteins (large subunit, LSU) in gray, ribosomal proteins (small subunit, SSU) in light green, maturation in red, membrane transport subunit in light blue, transfer RNAs in black, and ribosomal RNAs in pink. Arrows indicate the direction of gene transcription.

Annotation of the *I. domestica* mitochondrial genome revealed a total of 34 unique protein-coding genes, including 24 unique core mitochondrial genes and 10 non-core genes, along with 16 tRNA genes and 3 rRNA genes (**Table 3**; **Figure 3**). The annotation details could be found in the **Supplementary Note 1**.

**Table 3:** Coding genes of the mitochondrial genome.

Group of genes	Name of genes
ATP synthase	*atp*1, *atp*4, *atp*6, *atp*8, *atp*9(×2)
NADH dehydrogenase	*nad*1, *nad*2, *nad*3, *nad*4, *nad*4L, *nad*5, *nad*6, *nad*7, *nad*9
Cytochrome *b*	*cob*
Cytochrome *c* biogenesis	*ccm*B, *ccm*C, *ccm*FC, *ccm*FN
Cytochrome *c* oxidase	*cox*1, *cox*2, *cox*3
Maturases	*mat*R
Protein transport subunit	*mtt*B
Ribosomal protein large subunit	*rpl*2, *rpl*5, *rpl*16
Ribosomal protein small subunit	*rps*3, *rps*11, *rps*12, *rps*13, *rps*14, *rps*19
Succinate dehydrogenase	*sdh*4
Ribosome RNA	*rrn*5, *rrn*18, *rrn*26(×2)
Transfer RNA	*trn*C-GCA, *trn*D-GUC, *trn*E-UUC, *trn*F-GAA(×2), *trnf*M-CAU(×2), *trn*H-GUG, *trn*I-CAU, *trn*K-UUU, *trn*M-CAU, *trn*N-GUU, *trn*P-UGG, *trn*Q-UUG(×2), *trn*S-GCU, *trn*S-UGA, *trn*W-CCA, *trn*Y-GUA

**Figure 4:**
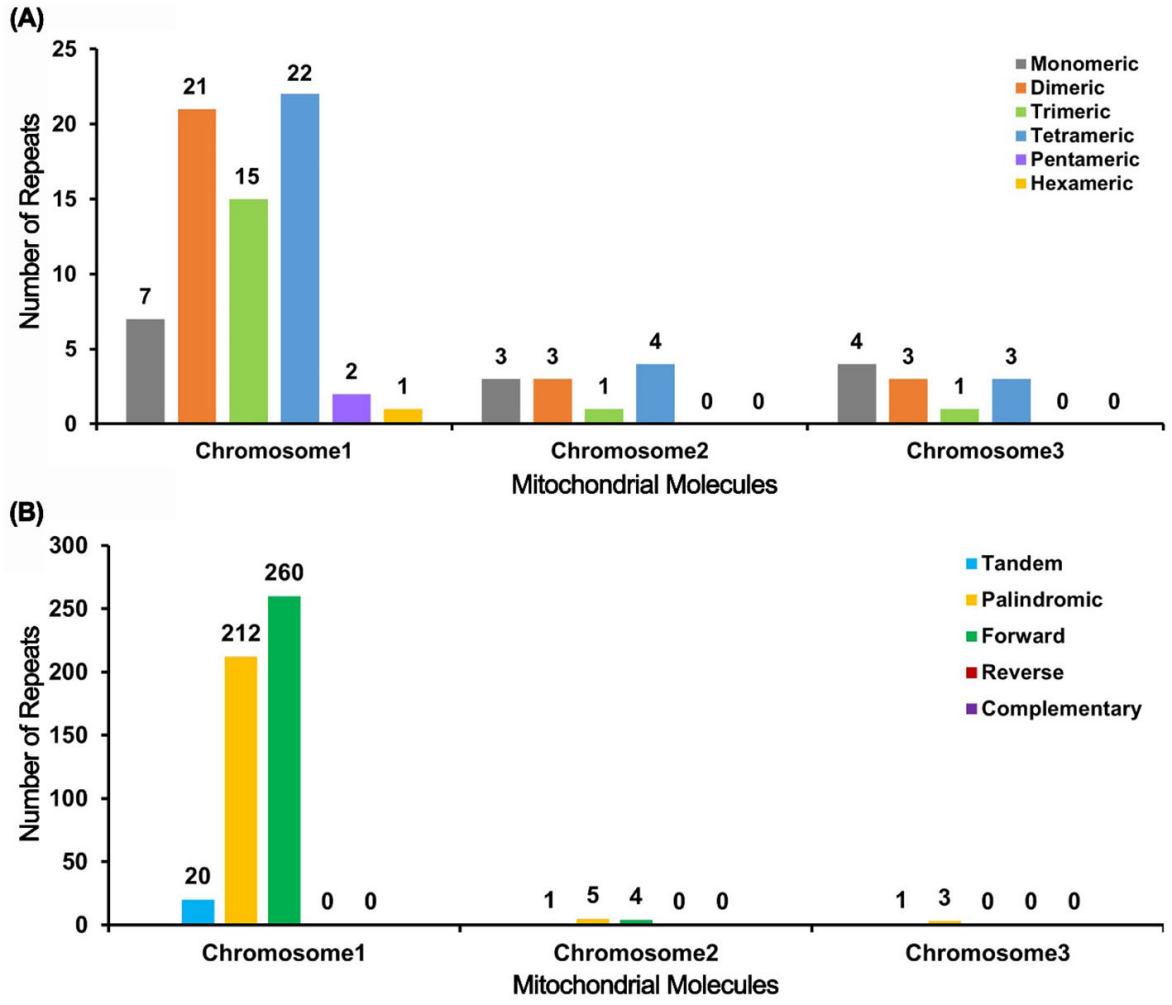
Bar Chart Analysis of Repeat Sequences. **(A)** The bar chart illustrates the distribution of simple sequence repeats (SSRs) across the mitochondrial molecules. The x-axis represents the different mitochondrial chromosomes (Chromosome 1, Chromosome 2, and Chromosome 3), while the y-axis indicates the number of repeat fragments identified. The color coding is as follows: gray bars represent monomer SSRs, orange bars represent dimer SSRs, green bars represent trimer SSRs, blue bars represent tetramer SSRs, purple bars represent pentamer SSRs, and yellow bars represent hexamer SSRs. **(B)** The bar chart shows the distribution of different types of repeat sequences within the mitochondrial genome. The x-axis represents the mitochondrial chromosomes (Chromosome 1, Chromosome 2, and Chromosome 3), and the y-axis denotes the number of repeat fragments observed. The color legend is defined as follows: blue bars indicate tandem repeats, yellow bars denote palindromic repeats, and green bars signify forward repeats.

The analysis of the *I. domestica* mitochondrial genome reveals significant variability in repeat sequence distribution across its chromosomes (**Supplementary Tables S1** **–** **S3**). Chromosome 1 exhibits the highest diversity, with 68 SSRs and 472 pairs of scattered repeats, including 212 palindromic and 260 forward repeats, indicating a complex genomic architecture. In contrast, Chromosomes 2 and 3 contain fewer SSRs (11 each) and fewer scattered repeat pairs. Chromosome 2 has 9 pairs, with a balanced distribution of 5 palindromic and 4 forward repeats, while Chromosome 3 features 3 pairs of repeats, all palindromic (**Figure 4**). These findings highlight the structural diversity within the mitochondrial genome, with potential implications for the genetic stability and functional adaptation of *B chinensis*.

### 3.4 Identification of MTPT

**Figure 5:**
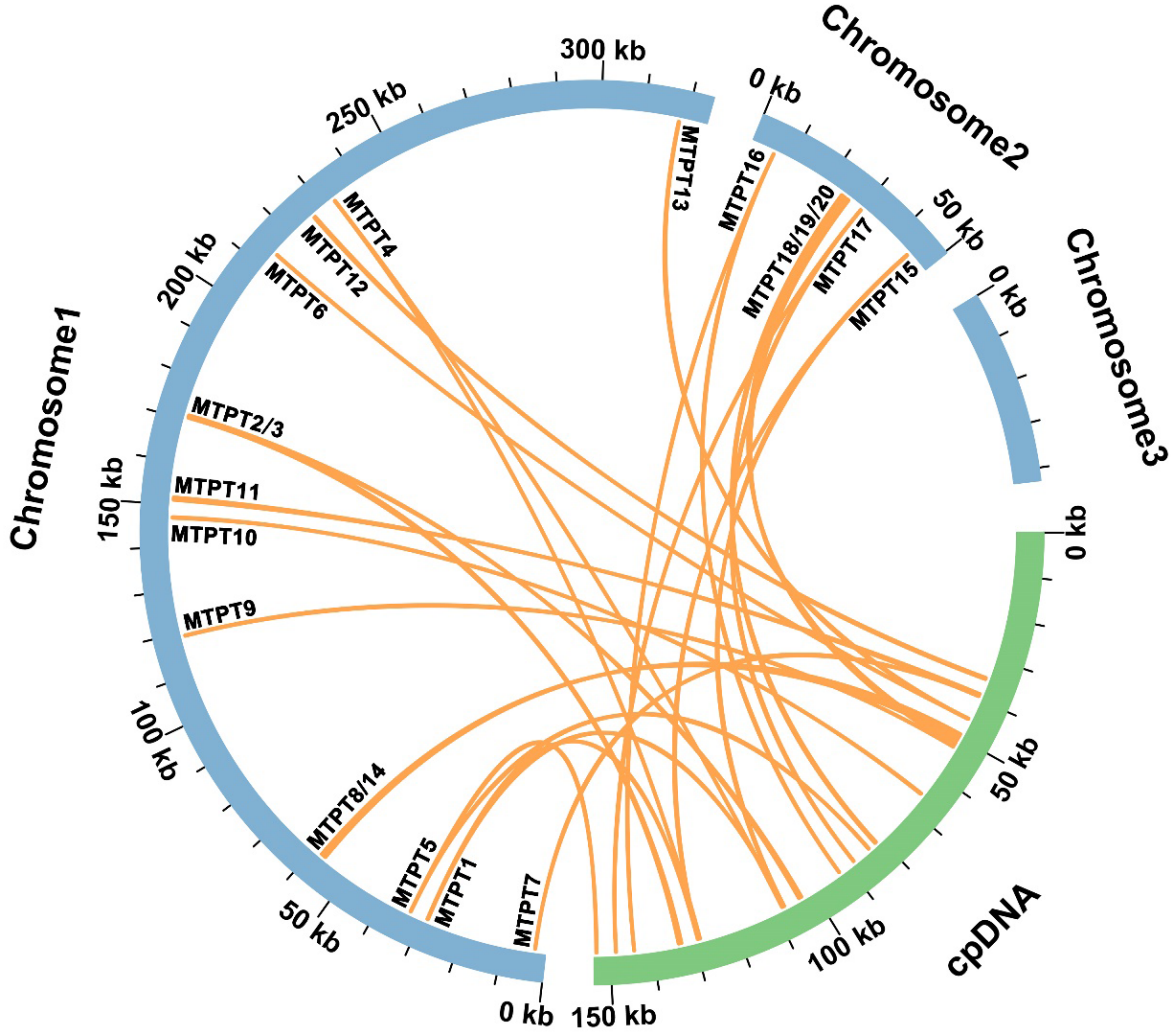
Sequence Migration Analysis. The diagram features blue arcs representing the mitochondrial genome and green arcs representing the plastome. Purple lines connecting the arcs indicate homologous segments between the genomes.

Based on sequence similarity analysis conducted in *I. domestica*, a total of twenty homologous fragments were identified between the organelle genomes, encompassing a cumulative length of 4,594 base pairs, which constitutes 1.10% of the mitochondrial genome (**Figure 5**; **Supplementary Table S4**). The longest of these fragments, designated MTPT14, spans 1,109 base pairs. Detailed annotation of these sequences uncovered three complete genes, all categorized as tRNA genes (*trnH-GUG*, *trnN-GUU*, *trnW-CCA*). A transfer hotspot is defined as a specific segment within mitochondrial plastid DNA (MTPT) that undergoes repeated transfer events during the migration process, indicating a high frequency of genetic exchange in this region. The regions MTPT1-5 and MTPT15-17 are identified as transfer hotspots, appearing as single copies within the mitochondrial genome and as duplicate copies in the inverted repeat (IR) sections of the chloroplast genome. Conversely, the remaining MTPT regions are uniformly present as single copies.

### 3.5 Phylogenetic analysis

**Figure 6:**
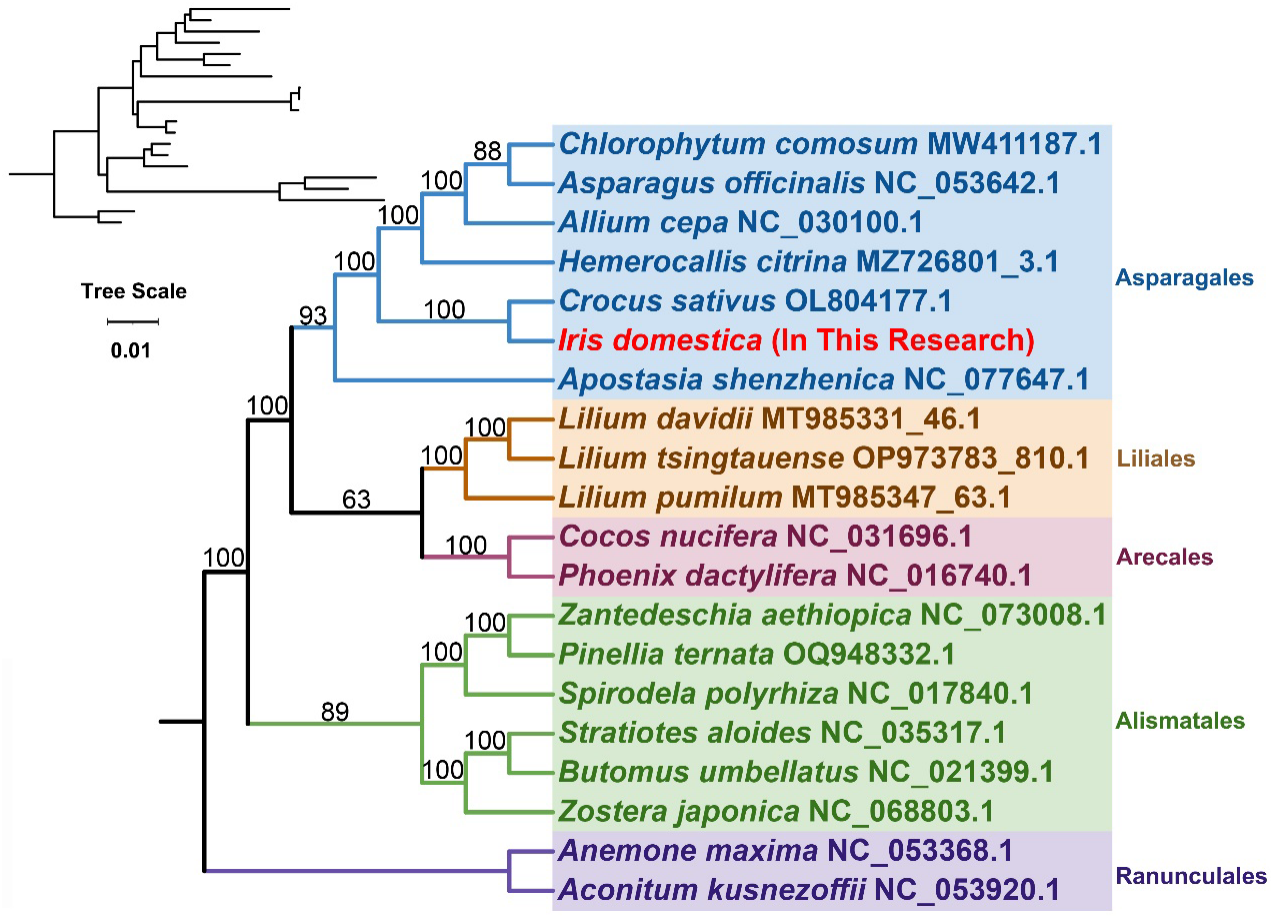
Phylogenetic Analysis. This figure illustrates the phylogenetic relationships derived from the analysis of genetic data, visually represented in a phylogenetic tree. On the left, a dendrogram displays the original topological structure without branch lengths, illustrating the cladistic relationships.

Based on the common genes (*atp1, atp4, atp6, atp8, atp9, ccmB, ccmC, ccmFC, ccmFN, cob, cox1, cox2, cox3, matR, mttB, nad1, nad2, nad3, nad4, nad4L, nad5, nad6, nad7* and *nad9*), the phylogenetic tree was built for 20 species across 5 orders of angiosperms. The details of plant species were provided in **Supplementary Table S5**. The details of the common genes used could be found in the **Supplementary Note 2**. Two species from the Ranunculales order were used as outgroups (**Figure 6**). The phylogenetic topology is consistent with the latest classification by the Angiosperm Phylogeny Group (APG IV). The species *I. domestica* belongs to the order Asparagales and the family Iridaceae, showing the closest phylogenetic relationship with *Crocus sativus*.

### 3.6 RNA editing site identification

**Figure 7:**
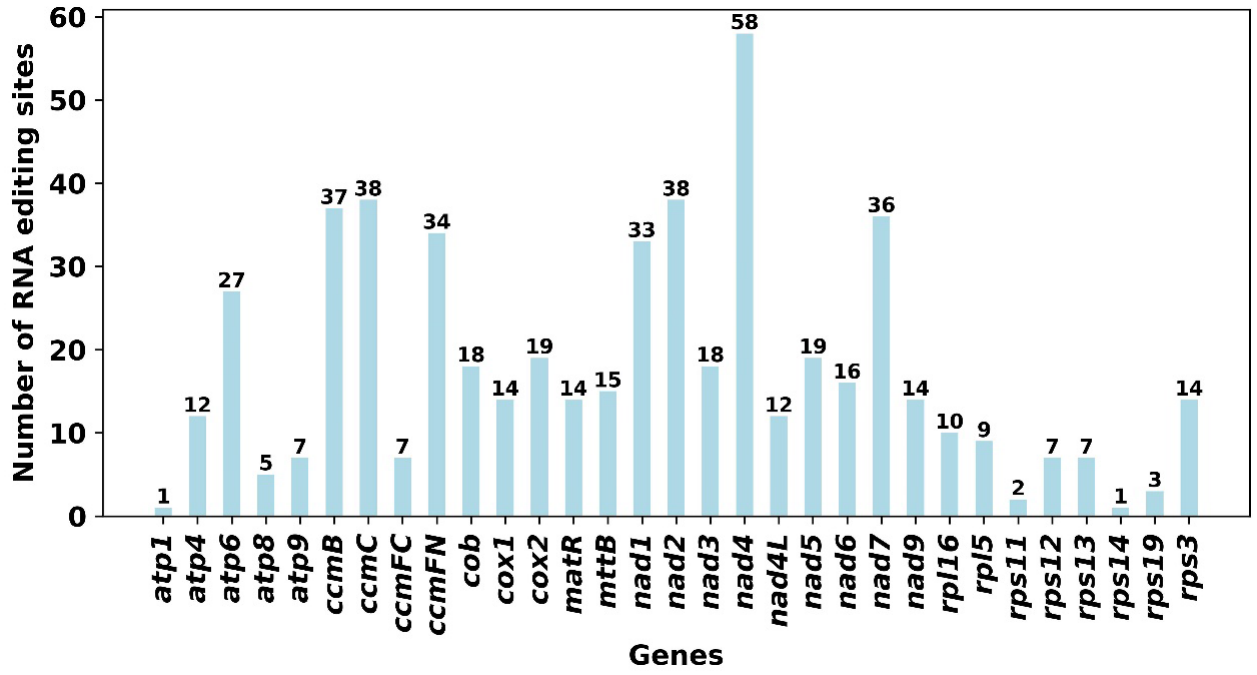
Predicted RNA Editing Sites in Mitochondrial PCGs. This figure displays the number of RNA editing sites predicted across various protein-coding genes (PCGs) in the mitochondrial genome. The data is presented in a bar chart format, providing a comparative view of RNA editing site distribution among the PCGs.

A total of 545 putative RNA editing sites were identified across 34 mitochondrial protein-coding genes (PCGs), with all modifications exclusively involving cytosine-to-uracil (C-to-U) conversions (**Figure 7**; **Supplementary Table S6**). The gene *nad4* demonstrated the highest frequency of RNA editing, with 58 identified sites. This was followed closely by *ccmC* and *nad2*, each exhibiting 38 editing sites. In contrast, minimal editing activity was observed in *atp1* and *rps14*, with only a single editing site in each, underscoring substantial variability in RNA editing frequency among different mitochondrial genes. In these 545 RNA editing sites, there are a total of 29 synonymous substitution sites and 516 nonsynonymous substitution sites.

In the 516 nonsynonymous RNA editing sites, four unique RNA editing sites were identified across different genes, each exhibiting notable alterations in the genetic code that may influence the start or stop codon. The gene *nad1* exhibits an editing event at base position 2, altering the codon from ACG to AUG and converting threonine (Thr) to methionine (Met) at the amino acid level. This site, surrounded by the motifs AGTGAATAGAAAATCGAAAA and GTACATTGCTGTTCCAGCGG, has a high editing frequency of 0.978. In atp6, the editing at base position 718 changes the codon CAA to UAA, replacing glutamine (Gln) with a stop codon (End), and is flanked by motifs ATGATGCTACAAATCTCCAT and AAAATGAGTCATTTCATAAT, with a probability of 0.983. Similarly, atp8 undergoes editing at base position 265, where the codon CAG is altered to UAG, also resulting in a stop codon, situated between motifs CTGAAGTATCCCAATGGTGT and AGACCGTCGCCTTCTTGGTA, showing a probability of 0.914. Lastly, the gene ccmFC features an edit at base position 811 from CGA to UGA, substituting arginine (Arg) for a stop codon, with motifs AGGATAAGTTGCATTGGGAT and GAGAAAGTTCCGTGGAGTTC on either side and an editing frequency of 0.994. These edits not only alter the amino acid sequence but also introduce premature termination codons, potentially affecting protein stability and function dramatically.

## 4 Discussion

### 4.1 Multichromosomal structure and recombination

Mitochondria tend to show more pronounced genomic embellishments compared to plastids. This observed pattern is likely due to the transfer of nuclear-encoded proteins between these organelles, which allows for the parallel development of similar genomic features within different cellular compartments (Smith and Keeling, 2015). Mitochondrial genomes of angiosperms exhibit extensive structural diversity. Beyond the single circular configuration, reminiscent of prokaryotic plasmids, some plant species possess linear or even multichromosomal mitochondrial structures (Møller et al., 2021; Wu et al., 2022; Wang et al., 2024a). The recent study has shown that diminished availability of homologous templates in plant mitochondrial DNA accelerates nucleotide substitution rates due to impaired repair efficiency, underscoring the critical role of homologous recombination in DNA damage repair in plants (Wang et al., 2024b). Research suggests that these complex structures may arise by repeats. The multichromosomal mitochondrial genome structure in *I. domestica*, which primarily consists of three circular segments. Although the mitochondrial genome of *Populus simonii* is twice the size of that of *I. domestica*, it similarly comprises three circular chromosome molecules (Bi et al., 2022). *P. simonii* contains no repeats longer than 350 bp, whereas in *I. domestica*, we detected two repeat sequences, R1 (7,784 bp) and R2 (3,519 bp), which cause recombination, generating two distinct substructures. In *Ipomoea batatas* mitochondrial genome, three repeat sequences (with lengths of 253, 78, and 62 bp) facilitate recombination, enabling a single circular chromosome molecule to rearrange into as many as four independent circular chromosome molecules (Yang et al., 2022). In *Scutellaria tsinyunensis*, a direct repeat of 175 bp allows the occasional formation of two independent circular chromosome molecules from a single circular chromosome (Li et al., 2021). Similarly, in *Panax notoginseng*, seven subgenomic rings share a conserved 852 bp segment (Yang et al., 2023). However, the mechanisms and products of these recombination processes remain insufficiently understood. For instance, it remains unclear whether additional recombination events or alternative repeat sequences contribute to structural variation in *I. domestica*. Furthermore, it is yet to be determined whether the R1 and R2 repeat sequences could concurrently mediate recombination to form a distinct, independent circular chromosome molecule.

### 4.2 Diversity of MTPTs

The phenomenon of mitochondrial plastid DNA transfer (MTPT) in plant genomes offers insights into gene migration and evolutionary adaptations across different species (Wang et al., 2007; Wang et al., 2018; Jiang et al., 2023). In *I. domestica*, 20 MTPTs were identified, comprising 1.10% of the mitochondrial genome. These fragments, especially MTPT1-5 and MTPT15-17, represent significant transfer hotspots. Comparative analysis with other species such as *Apostasia shenzhenica* (Ke et al., 2023) and *Asparagus officinalis* (Sheng et al., 2023) reveals a broader spectrum of gene migration. For example, *A. shenzhenica* shows a higher percentage (5.12%) of its mitochondrial genome derived from chloroplast DNA, indicating a more dynamic intergenomic exchange. In *A. officinalis*, 4.11% of the mitochondrial genome is chloroplast-derived, with significant portions of this DNA involving essential genes for ATP synthesis and rRNA. The onion (*Allium cepa*) (Kim et al., 2013) provides a contrast, showing a lower frequency of large transfer events but maintaining functionality in genes such as *rps19* and *rpl23*, which are integral to protein synthesis. This comparative approach helps delineate the adaptive strategies of different plant species in response to their unique ecological and evolutionary contexts. Further studies are needed to explore the mechanisms behind gene selection and retention during these transfer events, which will enhance our understanding of plant genomic evolution.

### 4.3 RNA editing events

Comparing these results with studies of related species, *Hemerocallis citrina* (Zhang et al., 2022) predicted 679 RNA editing sites across all 45 PCGs, indicating significant variations in the total number and distribution of RNA editing sites across species, which may reflect species-specific metabolic and functional needs. For example, *A. shenzhenica* (Ke et al., 2023) showed fewer predicted sites (416), but like our study, it exhibited a higher number of edits in complex I and cytochrome c biogenesis genes, consistent with the high editing frequencies observed in *ccmC* and *nad2* in our analysis. The study of *A. officinalis* (Sheng et al., 2023) paralleled our findings, with *nad4* having the most editing sites and *rps7* the fewest.

These comparative analyses underscore the importance of RNA editing in maintaining mitochondrial gene expression and functional stability, likely reflecting adaptive evolutionary responses to specific ecological environments. The frequent RNA editing events observed in genes like *nad4* may indicate their critical role in cellular energy metabolism. Collectively, RNA editing is not only a crucial aspect of gene expression regulation but also a key mechanism in plant adaptive evolution. Future research should further explore how RNA editing affects plant adaptive traits under different environmental conditions and how these editing events are selected and conserved.

In the study at hand, multiple RNA editing sites have been identified within the nad1 gene, which encodes a subunit of the NADH dehydrogenase complex, critical for mitochondrial electron transport and energy production. Specifically, the MEF25 protein in Arabidopsis, classified as an E+-subclass pentatricopeptide repeat (PPR) protein, precisely targets the RNA editing site at nad1-308 (Arenas-M et al., 2013). This specificity underscores the need for multiple editing factors to manage closely located targets within the nad1 transcript, as evidenced by the independent editing of an adjacent site at nad1-307 by different factors. Moreover, in maize, the E-subgroup PPR protein DEK55 impacts RNA editing across multiple sites in 14 mitochondrial gene transcripts, including nad1 (Ren et al., 2020). The alterations at these sites correlate with the malfunction of mitochondrial complex I and consequent abnormal kernel development. Given the presence of numerous editing sites on nad1 and their potential implications for mitochondrial functionality, the effects of these RNA editing sites on the broader genome functionality necessitate further detailed investigation. This research could elucidate the complex interactions within mitochondrial genes and their overarching impact on plant development and vitality.

## 5 Conclusions

We achieved the complete mitochondrial genome of *I. domestica*, providing the first detailed insights into its structural organization, gene content, and evolutionary adaptations. The multichromosomal architecture observed and characterized by three single chromosomes and extensive repeat sequences, underscores the structural complexity typical of plant mitochondrial genomes. Our findings of significant variability in repeat elements across chromosomes, as well as intergenomic transfers of plastid DNA, suggest that these features may play crucial roles in maintaining genome stability, gene regulation, and adaptation to environmental pressures.

## Data Availability

The mitochondrial genome data of Belamcanda chinensis used in this study are available in public repositories. The raw sequencing data can be accessed from the NCBI Sequence Read Archive (SRA) under accession numbers SRR31023088 (Illumina sequencing) and SRR31023089 (PacBio sequencing). The assembled mitochondrial chromosomes are available under GenBank accession numbers PQ468093 (chromosome 1), PQ468094 (chromosome 2), and PQ468095 (chromosome 3). Additional project information can be found in the BioProject PRJNA1172909 and BioSample SAMN44283668.
