# Supplementary figures and images for: Comprehensive analysis of the mitochondrial genome of Iris domestica emphasizing multichromosomal organization and repeat-mediated homologous recombination
Source: Front Plant Sci. 2025 Feb 27;15:1520033. doi: 10.3389/fpls.2024.1520033 (PMC11903213; doi:10.3389/fpls.2024.1520033)

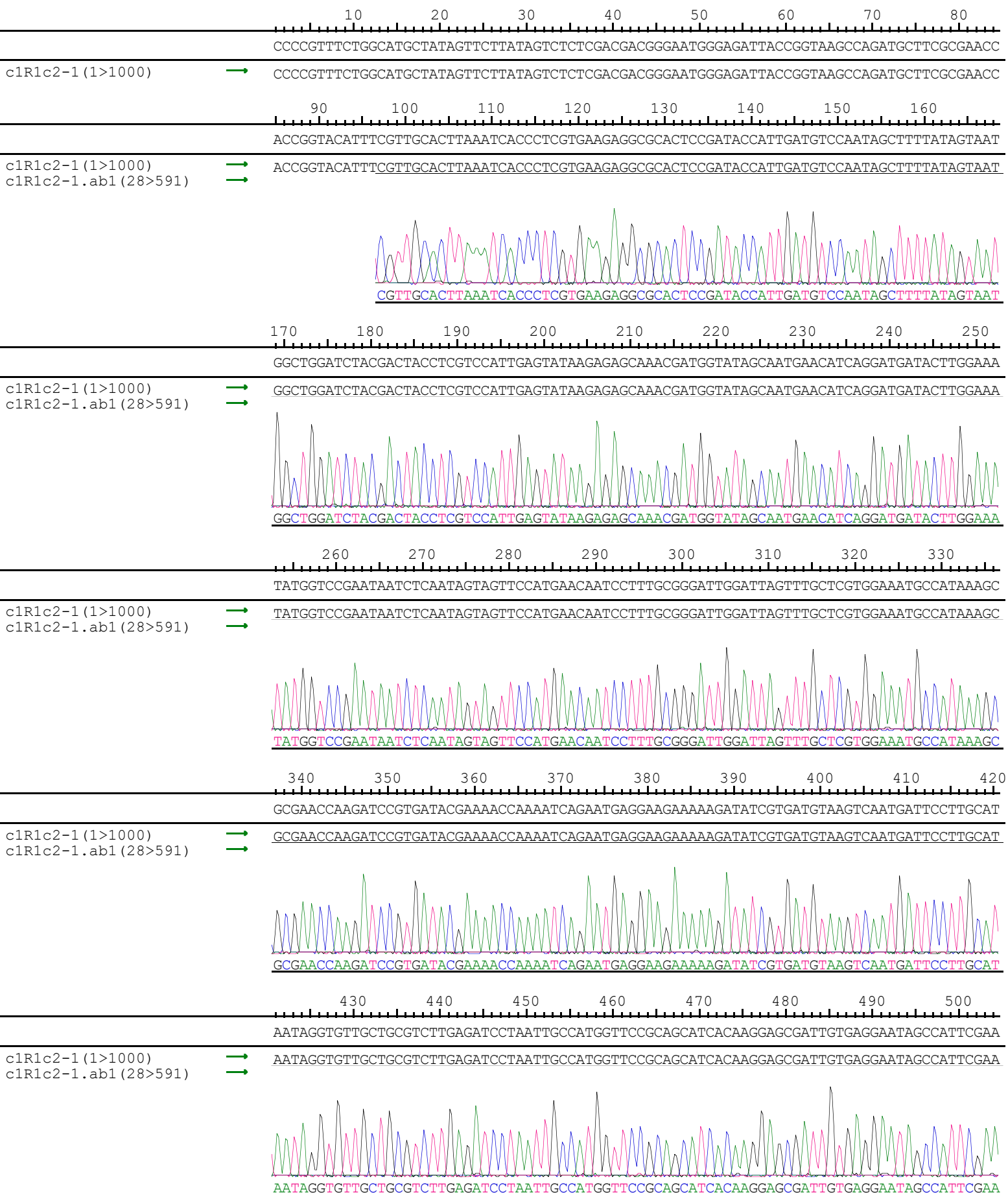

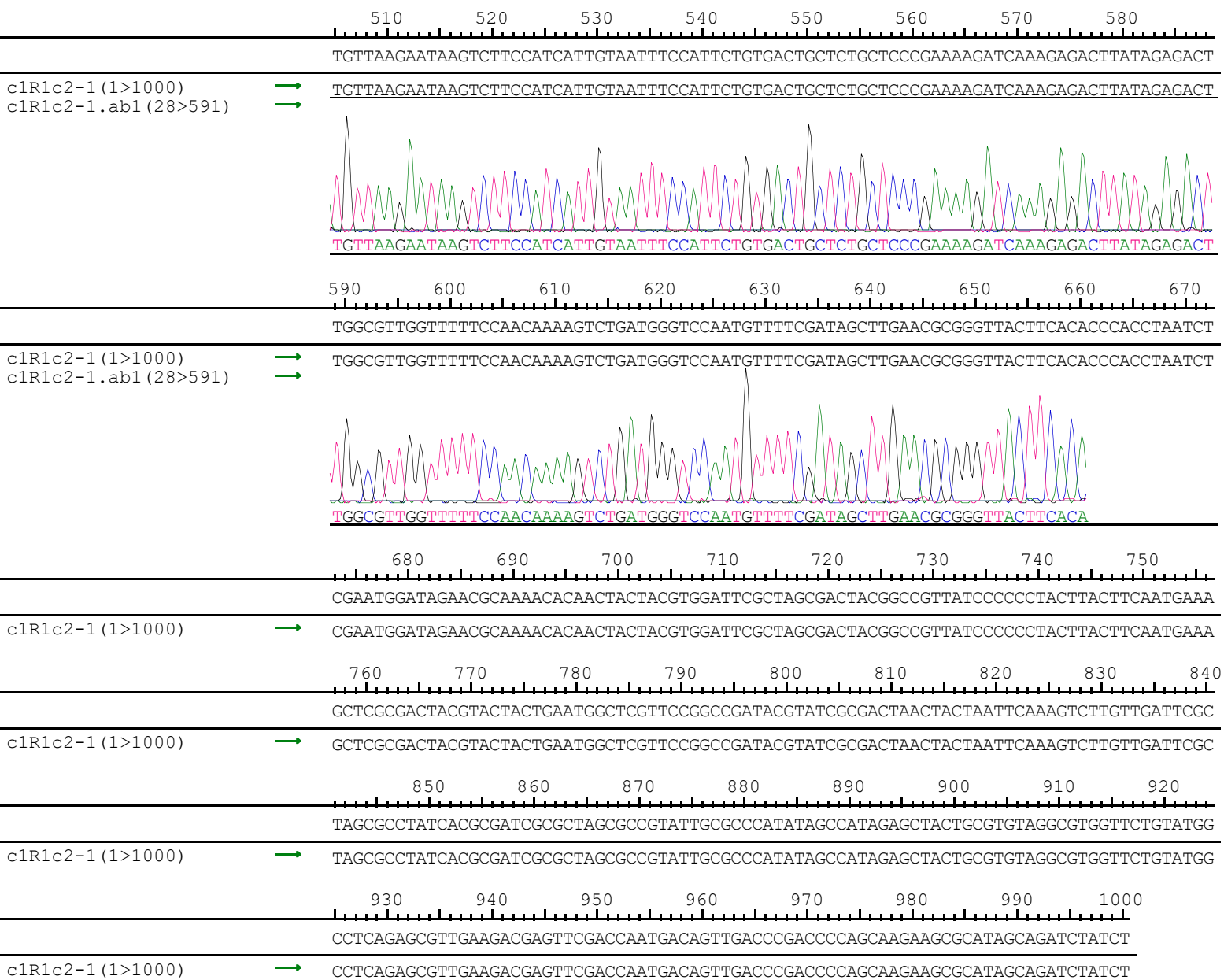

Supplement: Supplementary file 1 [file Presentation1.zip › Supplementary file 1 c1R1c2-1.pdf]

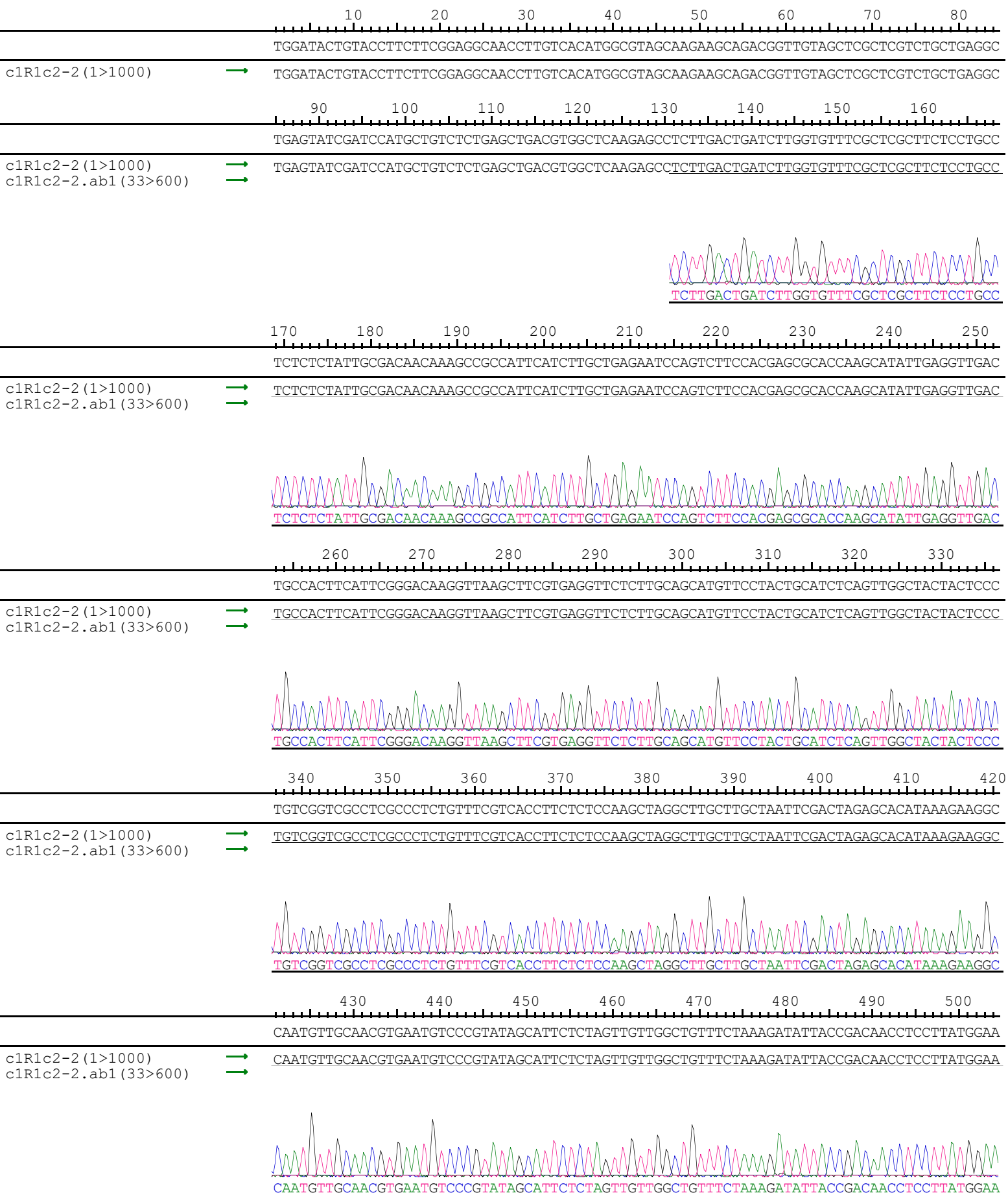

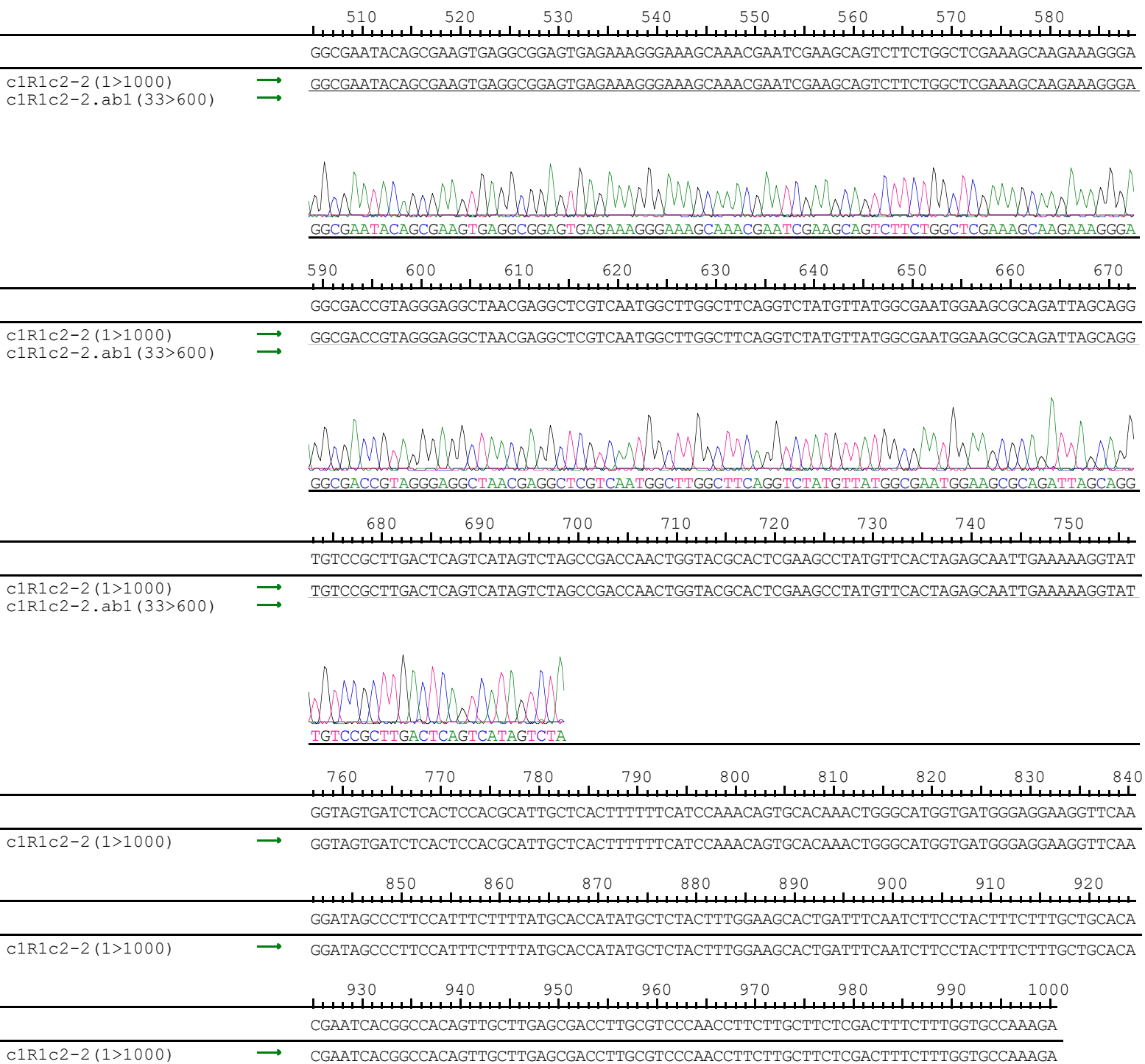

Supplement: Supplementary file 1 [file Presentation1.zip › Supplementary file 2 c1R1c2-2.pdf]

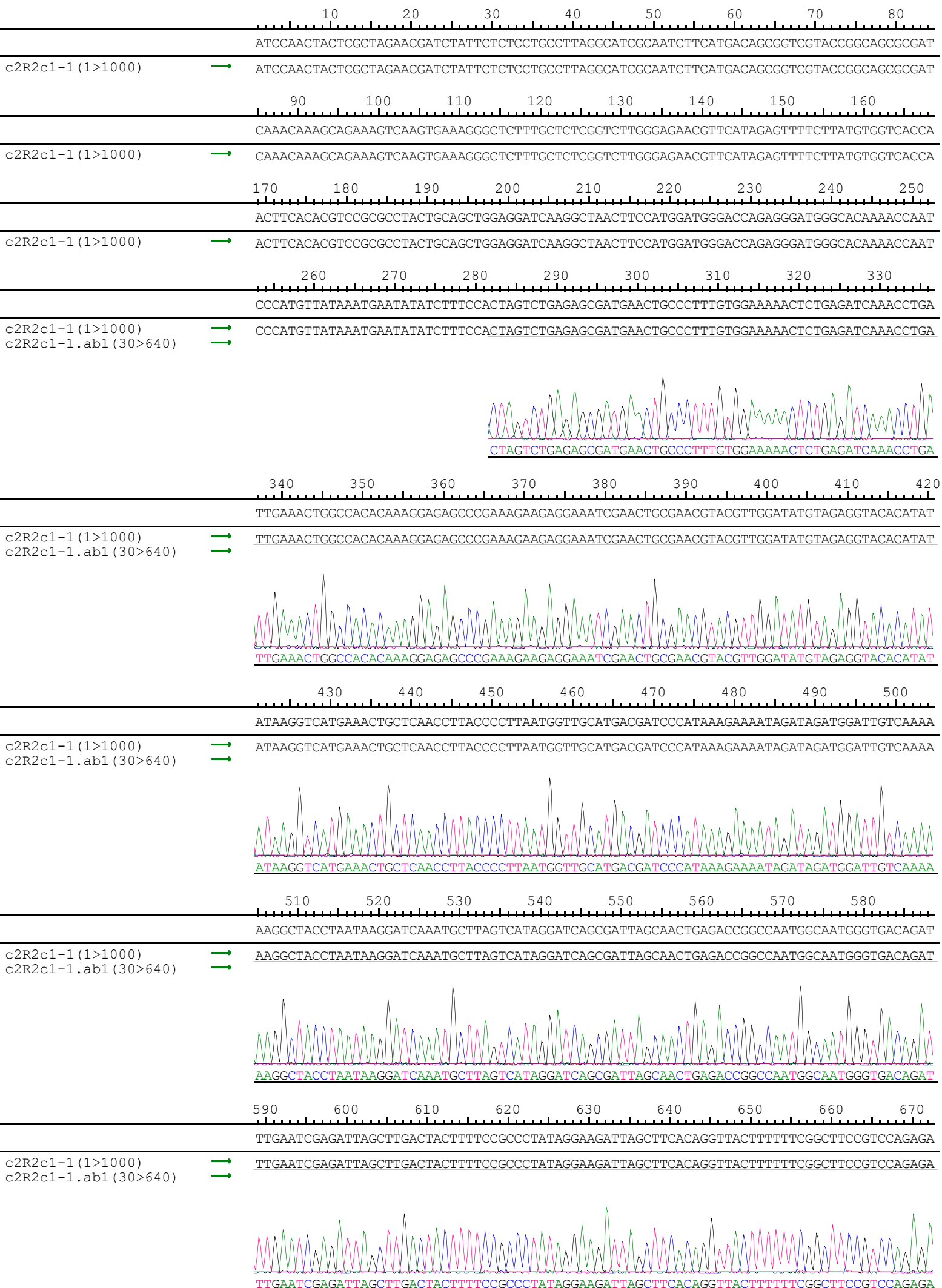

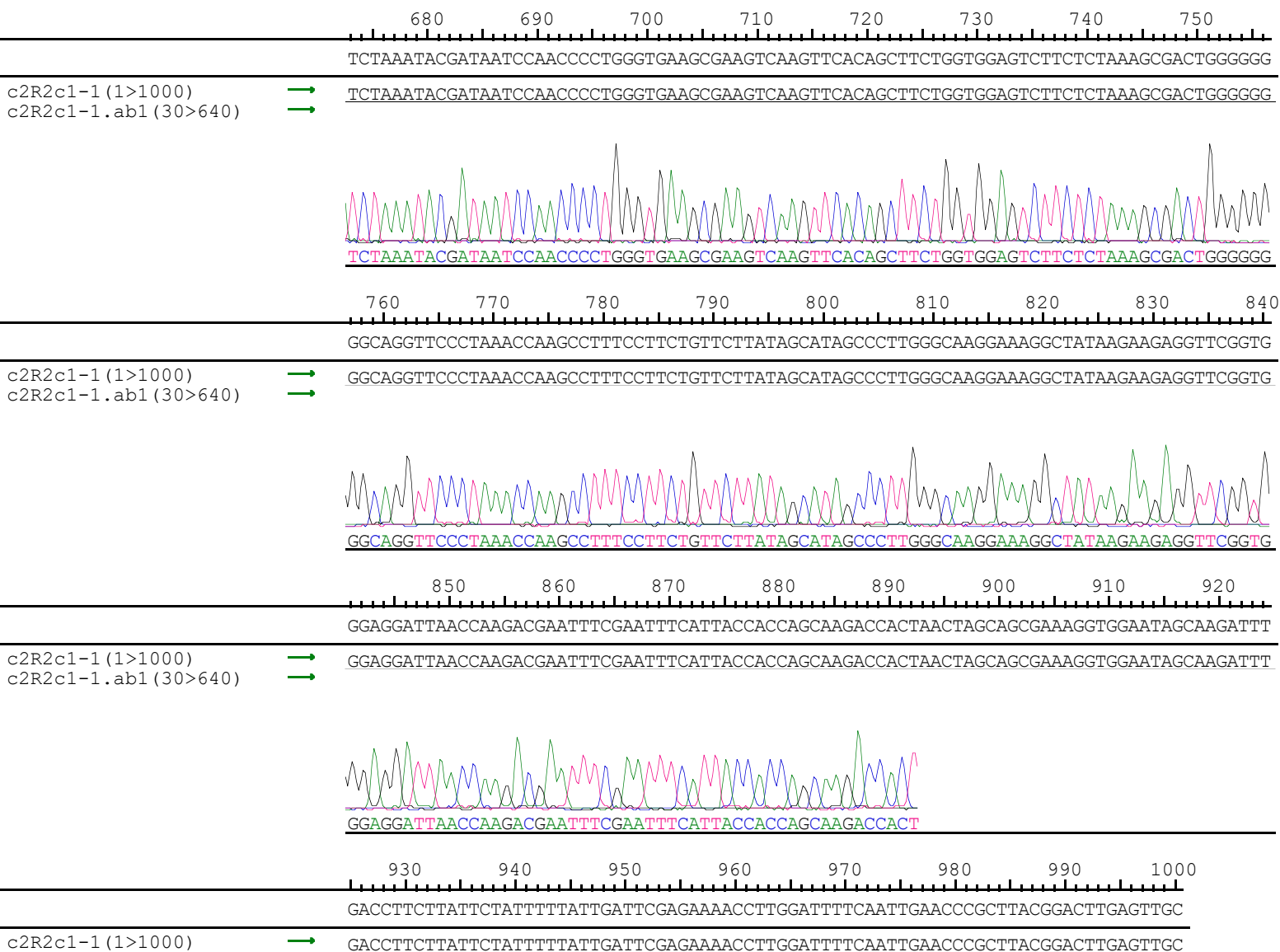

Supplement: Supplementary file 1 [file Presentation1.zip › Supplementary file 3 c2R2c1-1.pdf]

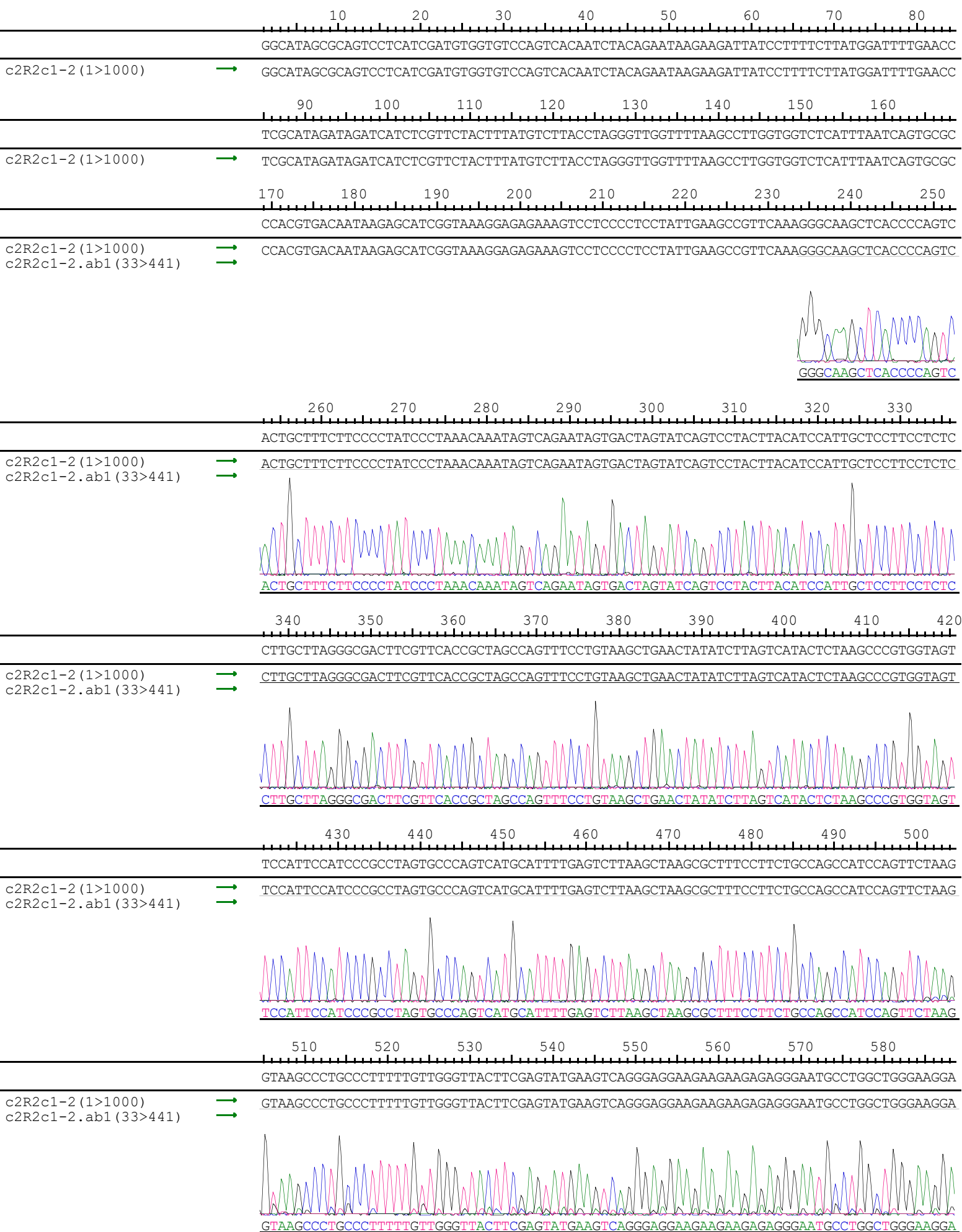

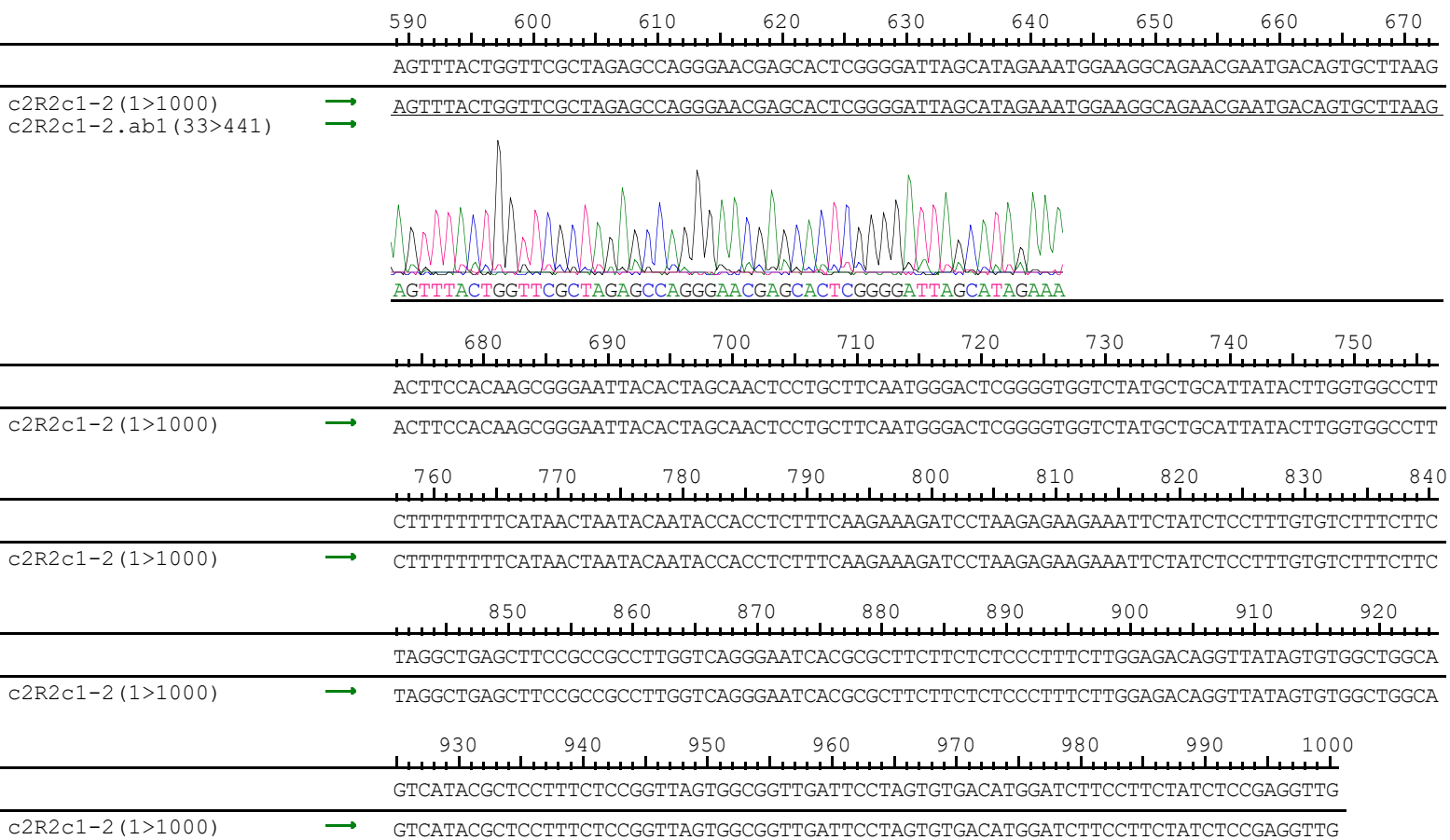

Supplement: Supplementary file 1 [file Presentation1.zip › Supplementary file 4 c2R2c1-2.pdf]

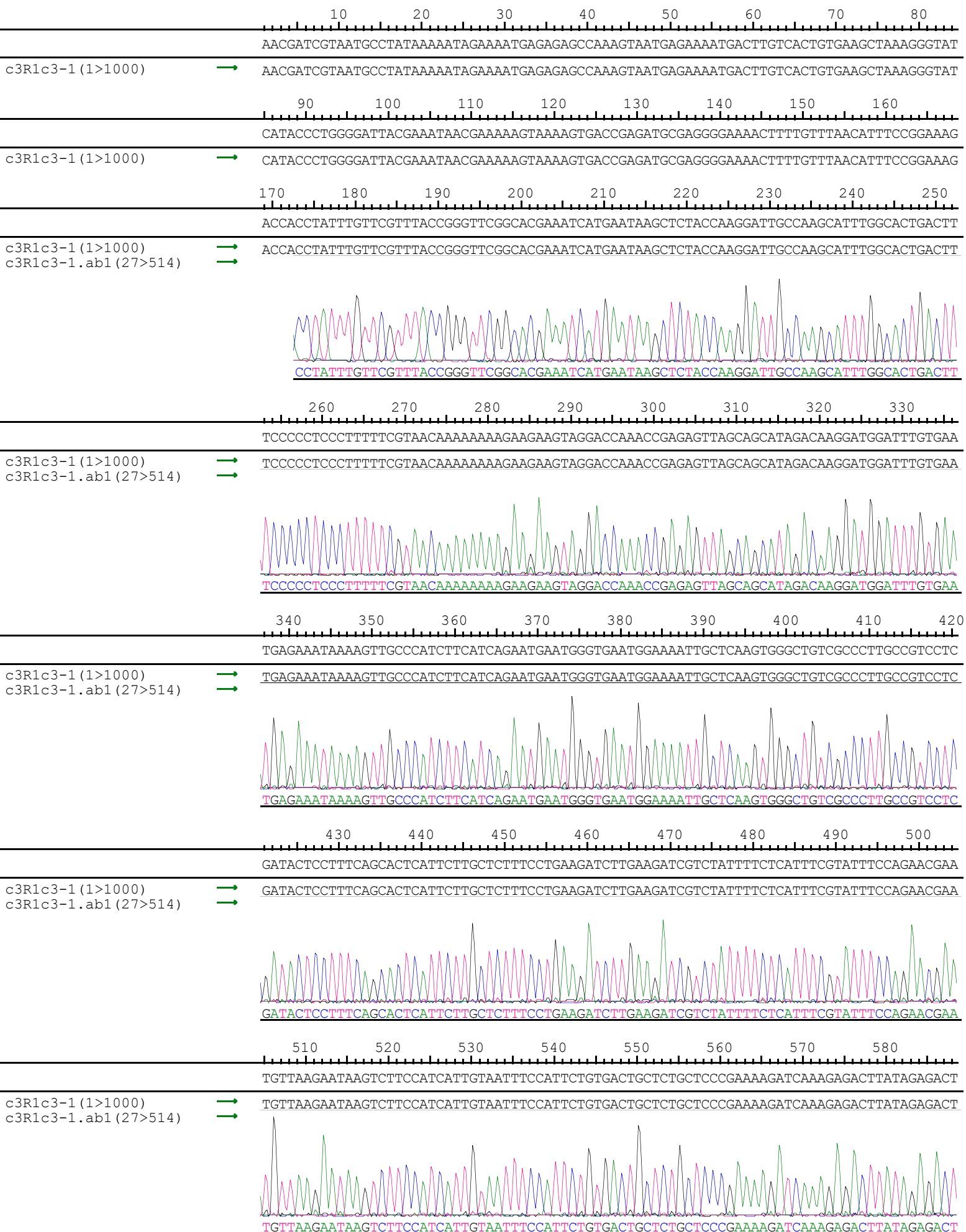

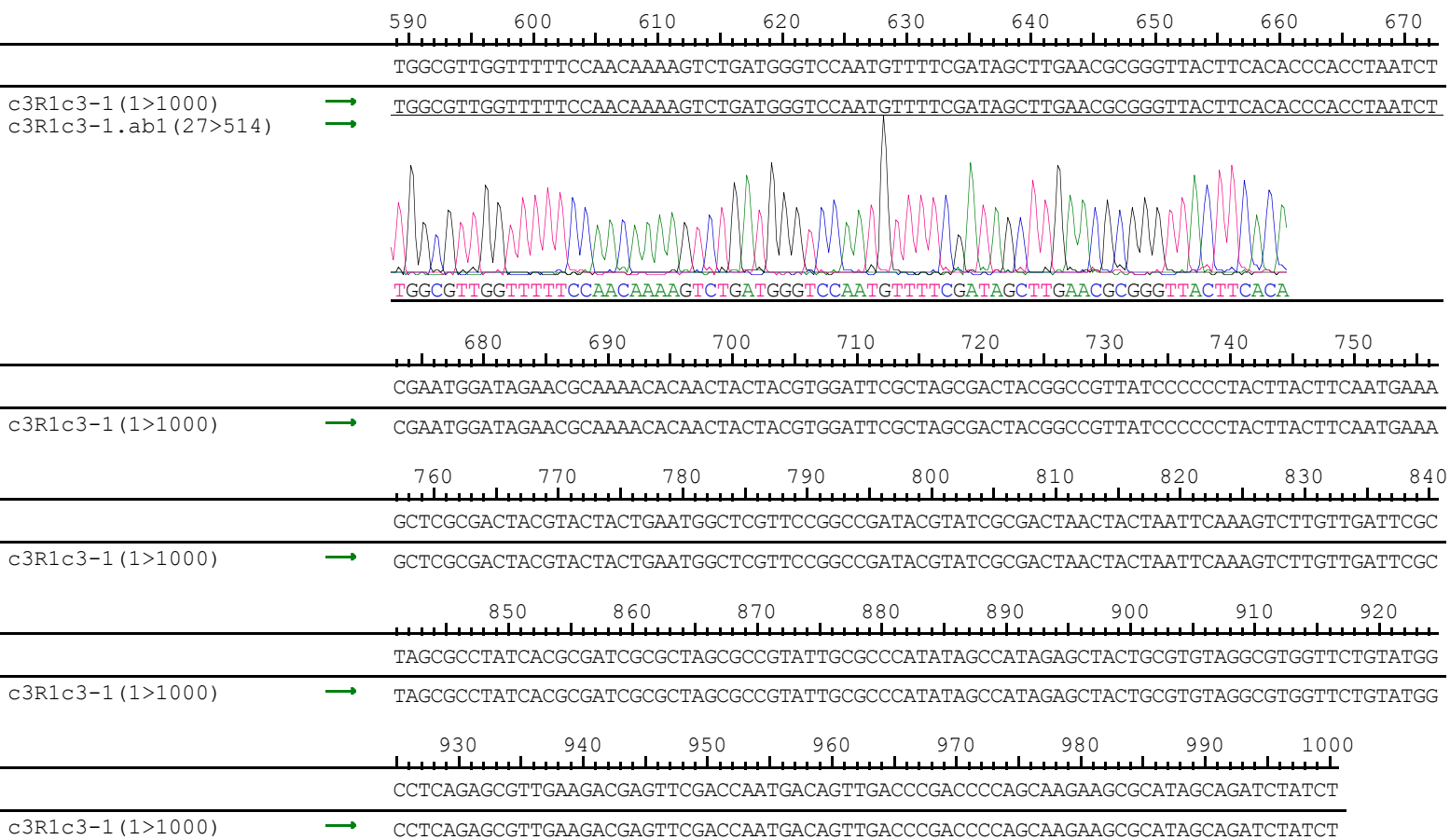

Supplement: Supplementary file 1 [file Presentation1.zip › Supplementary file 5 c3R1c3-1.pdf]

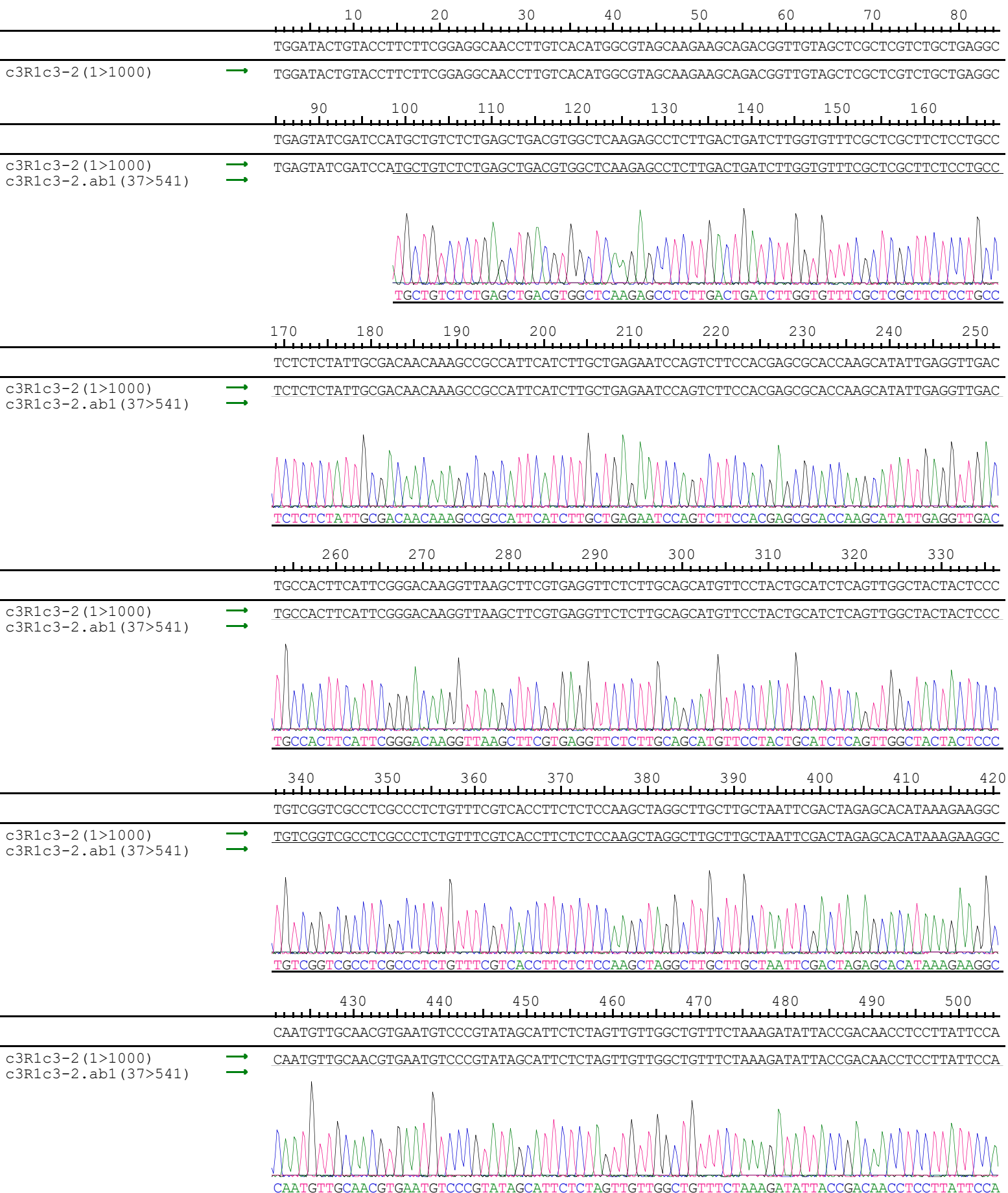

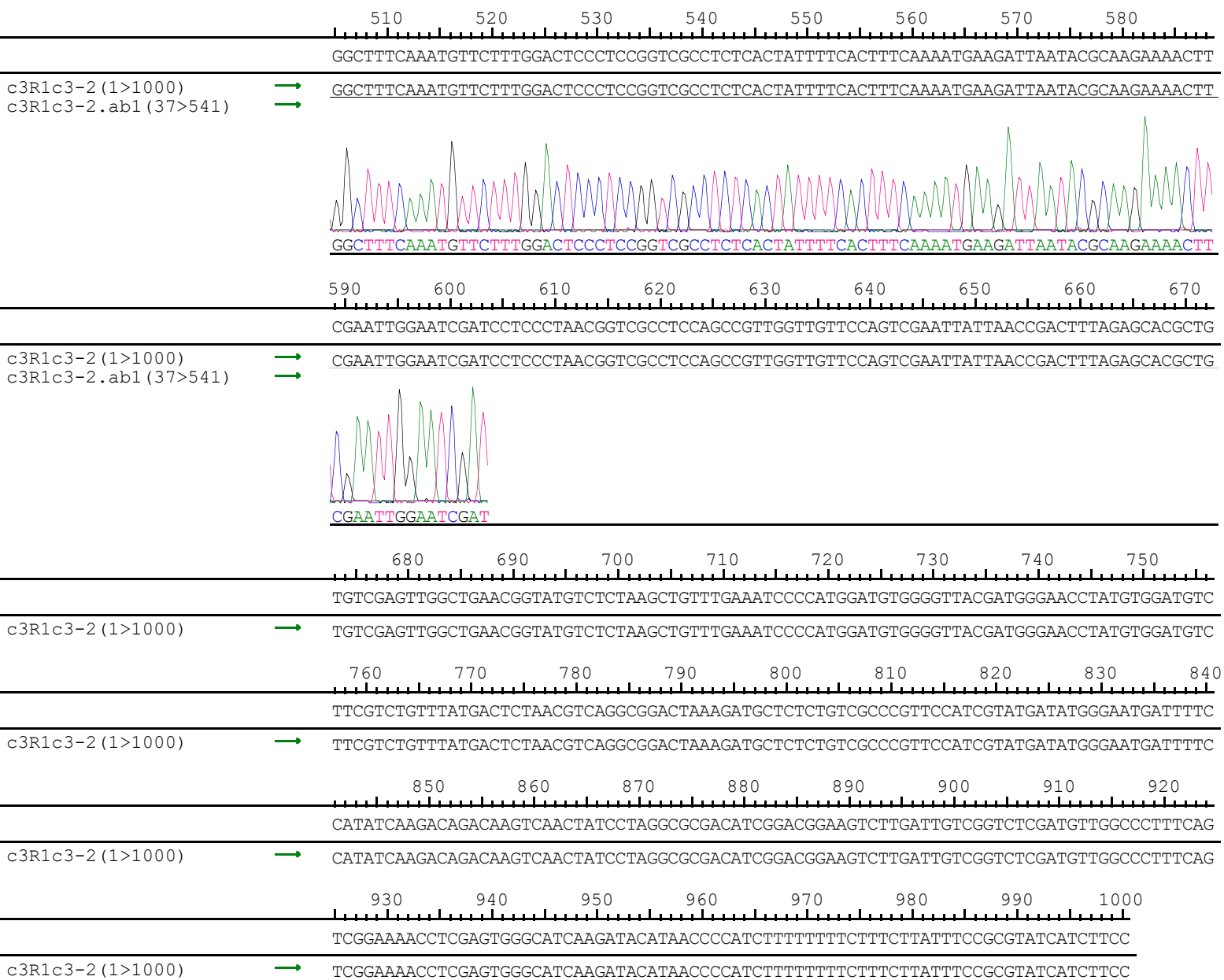

Supplement: Supplementary file 1 [file Presentation1.zip › Supplementary file 6 c3R1c3-2.pdf]

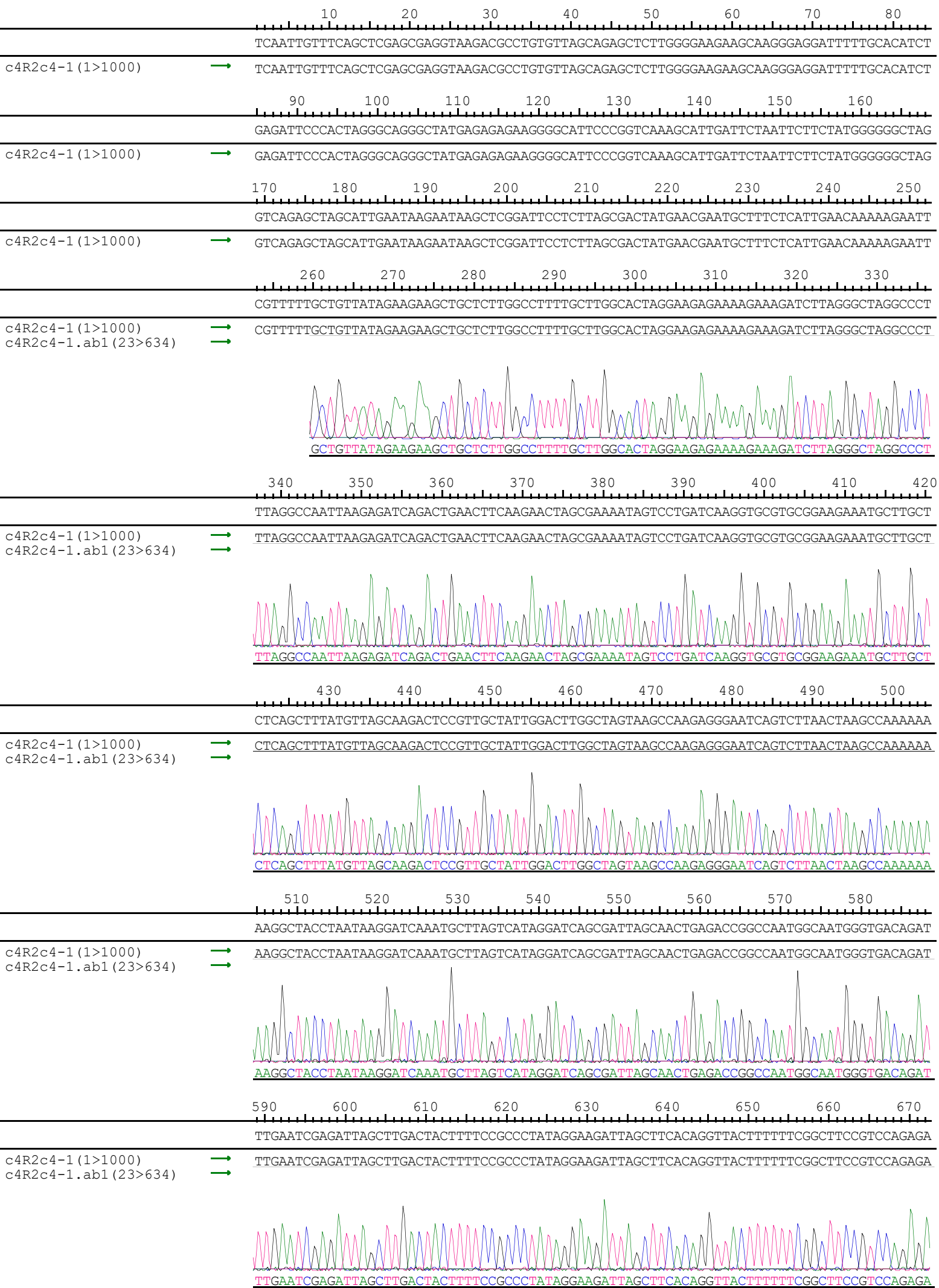

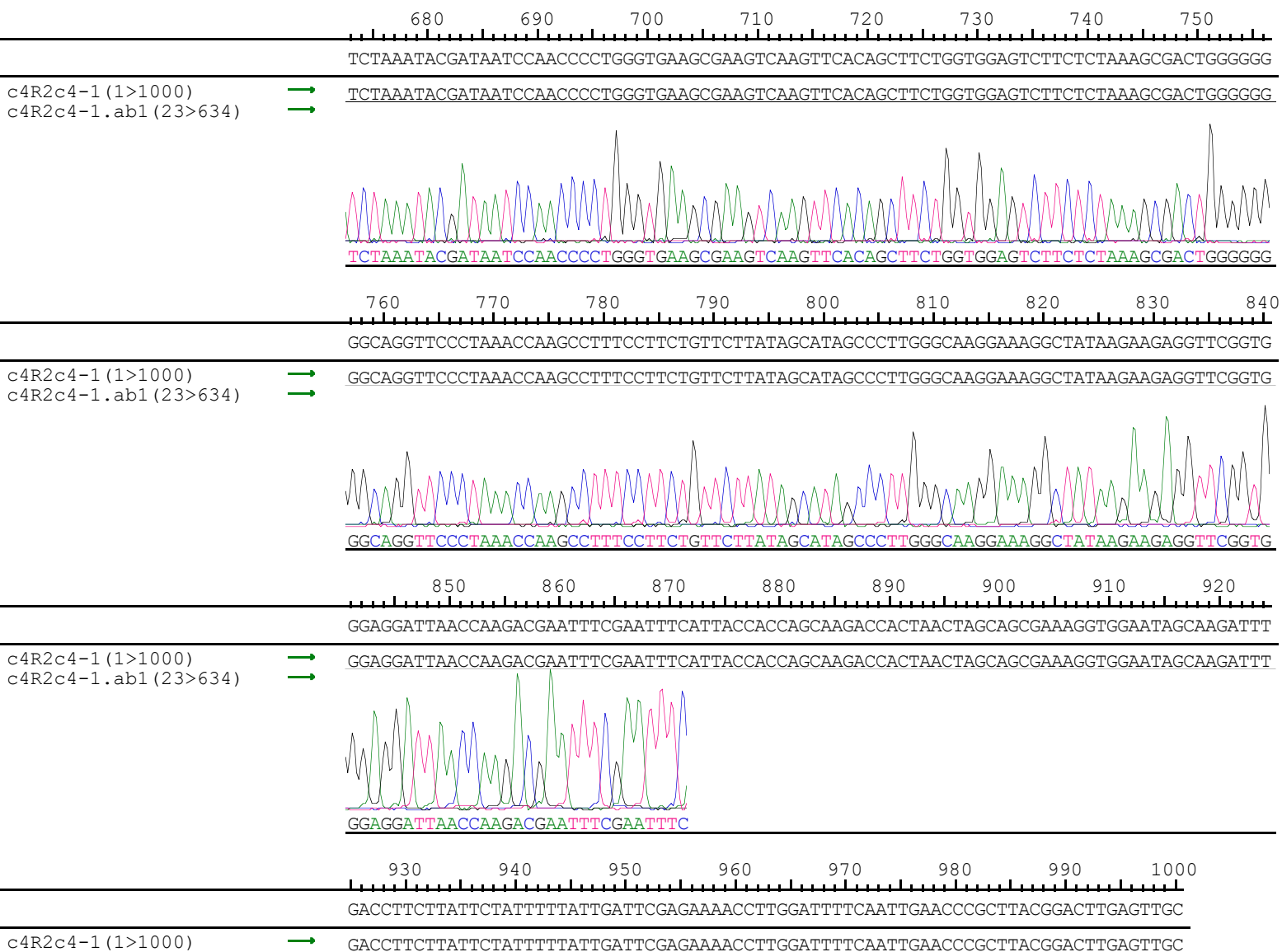

Supplement: Supplementary file 1 [file Presentation1.zip › Supplementary file 7 c4R2c4-1.pdf]

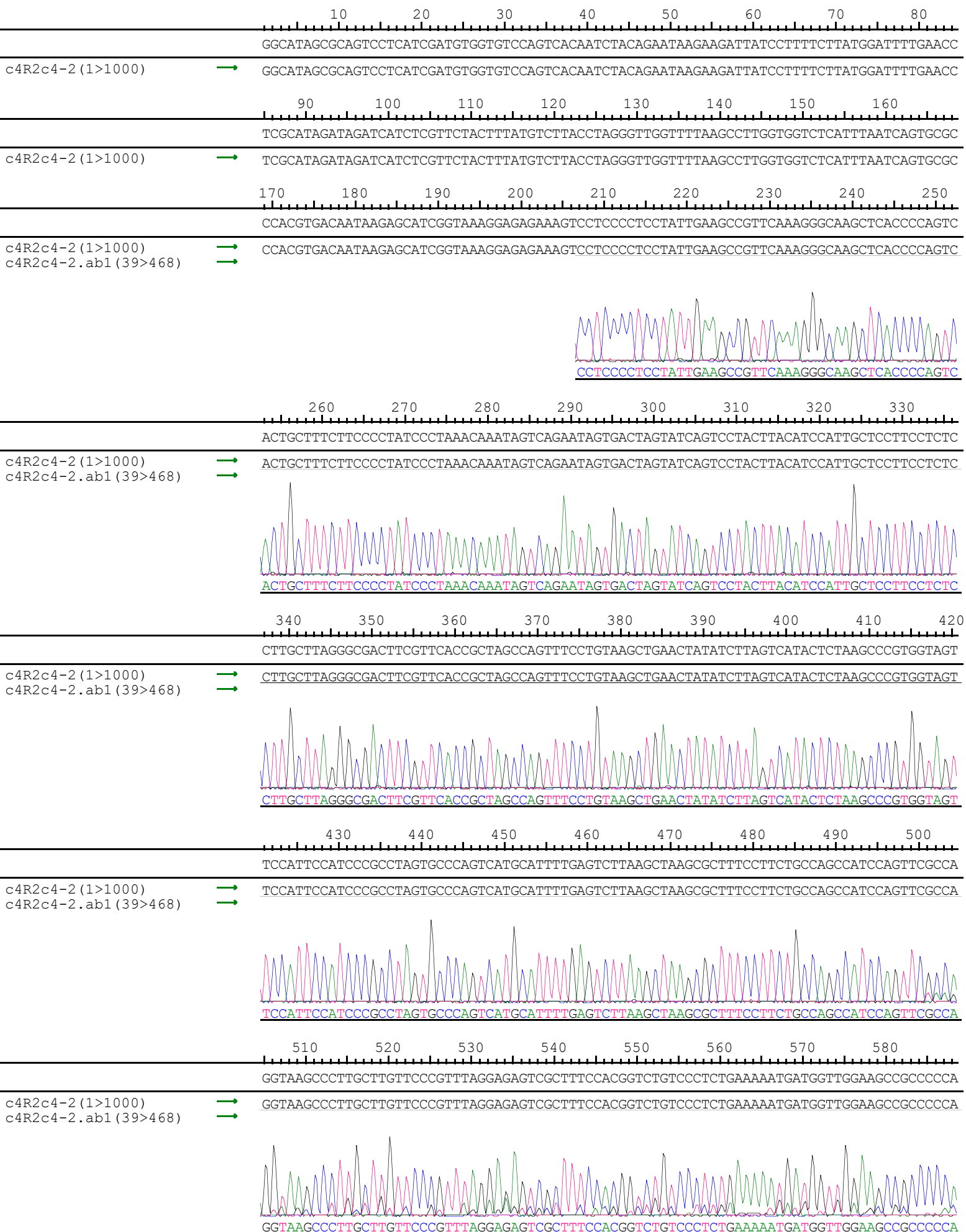

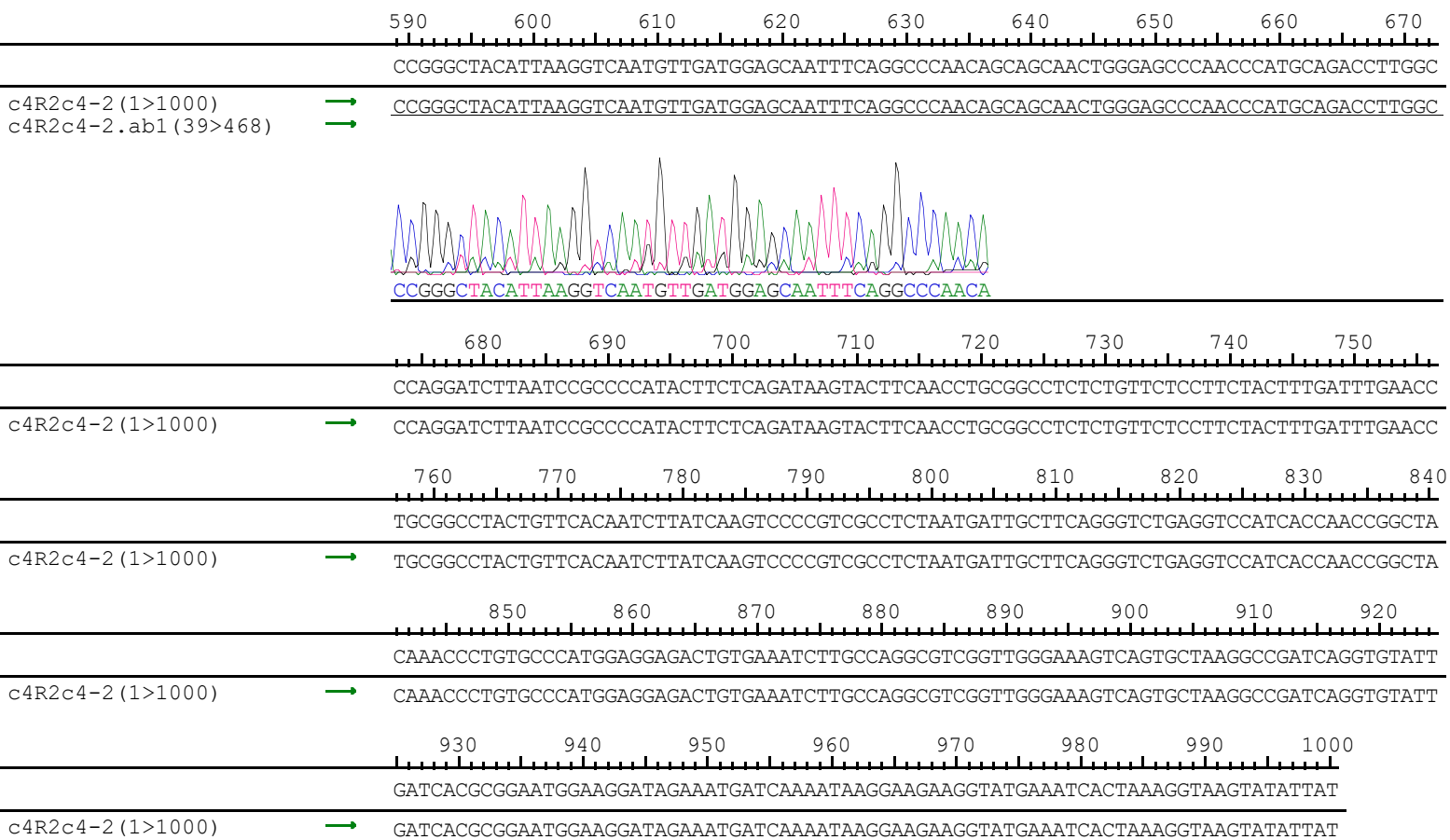

Supplement: Supplementary file 1 [file Presentation1.zip › Supplementary file 8 c4R2c4-2.pdf]
