# Supplementary material for: Comprehensive analysis of the mitochondrial genome of Iris domestica emphasizing multichromosomal organization and repeat-mediated homologous recombination
Source: Front Plant Sci. 2025 Feb 27;15:1520033. doi: 10.3389/fpls.2024.1520033 (PMC11903213; doi:10.3389/fpls.2024.1520033)
Supplement: Supplementary file 2 [file Presentation2.pdf]

Supplementary Note 1

The core genes include 5 ATP synthase genes (*atp1*, *atp4*, *atp6*, *atp8*, and *atp9*), 9 NADH dehydrogenase genes (*nad1*, *nad2*, *nad3*, *nad4*, *nad4L*, *nad5*, *nad6*, *nad7*, and *nad9*), 4 cytochrome C biogenesis genes (*ccmB*, *ccmC*, *ccmFC*, and *ccmFN*), 3 cytochrome C oxidase genes (*cox1*, *cox2*, and *cox3*), 1 membrane transport protein gene (*mttB*), 1 maturase gene (*matR*), and 1 ubiquinol-cytochrome C reductase gene (*cob*). The non-core genes include 3 large ribosomal subunit genes (*rpl2*, *rpl5*, *rpl16*), 6 small ribosomal subunit genes (*rps3*, *rps11*, *rps12*, *rps13*, *rps14*, *rps19*), and 1 succinate dehydrogenase gene (*sdh4*).

Supplementary Note 2

The protein-coding genes included are *atp1*, *atp4*, *atp6*, *atp8*, *atp9*, *ccmB*, *ccmC*, *ccmFC*, *ccmFN*, *cob*, *cox1*, *cox2*, *cox3*, *matR*, *mttB*, *nad1*, *nad2*, *nad3*, *nad4*, *nad4L*, *nad5*, *nad6*, *nad7*, and *nad9*.
